# Multidisciplinary investigation links backward-speech trait and working memory through genetic mutation

**DOI:** 10.1038/srep20369

**Published:** 2016-02-03

**Authors:** Stefan Prekovic, Dušica Filipović Đurđević, Gábor Csifcsák, Olivera Šveljo, Oliver Stojković, Milica Janković, Katarina Koprivšek, Laura E Covill, Milos Lučić, Thomas Van den Broeck, Christine Helsen, Fabiola Ceroni, Frank Claessens, Dianne F Newbury

**Affiliations:** 1 Department of Cellular and Molecular Medicine, Faculty of Medicine, KU Leuven, Leuven, Belgium; 2 Department of Psychology, Faculty of Philosophy, University of Novi Sad, Novi Sad, Serbia; 3 Laboratory for Experimental Psychology, Faculty of Philosophy, University of Belgrade, Belgrade, Serbia; 4 Institute of Psychology, Faculty of Arts, University of Szeged, Szeged, Hungary; 5 Diagnostic Imaging Center, Oncology Institute of Vojvodina, Sremska Kamenica, Serbia; 6 Faculty of Technical Sciences, University of Novi Sad, Novi Sad, Serbia; 7 Faculty of Medicine, University of Belgrade, Belgrade, Serbia; 8 Medical Faculty, University of Novi Sad, Novi Sad, Serbia; 9 Wellcome Trust Center for Human Genetics, University of Oxford, Oxford, UK. OX3 7BN

## Abstract

Case studies of unusual traits can provide unique snapshots of the effects of modified systems. In this study, we report on an individual from a Serbian family with the ability to rapidly, accurately and voluntarily speak backwards. We consider psychological, neural and genetic correlates of this trait to identify specific relevant neural mechanisms and new molecular pathways for working memory and speech-related tasks. EEG data suggest that the effect of word reversal precedes semantic integration of visually presented backward-words, and that event-related potentials above the frontal lobe are affected by both word reversal and the maintenance of backward-words in working memory. fMRI revealed that the left fusiform gyrus may facilitate the production of backward-speech. Exome sequencing identified three novel coding variants of potential significance in the *RIC3*, *RIPK1* and *ZBED5* genes. Taken together, our data suggest that, in this individual, the ability to speak backwards is afforded by an extraordinary working memory capacity. We hypothesise that this is served by cholinergic projections from the basal forebrain to the frontal cortex and supported by visual semantic loops within the left fusiform gyrus and that these neural processes may be mediated by a genetic mutation in *RIC3*; a chaperone for nicotinic acetylcholine receptors.

Working memory (WM) is a limited capacity system involved in the transient storage and processing of information, which is important for learning, reasoning and comprehension. Although the model and components of WM are still debated, there is a consensus that WM is essential for the development of many language-related traits including speech production and processing and language learning^1^. Evidence suggests that, in neurological terms, memory is a distributed function, but the prefrontal cortex (ventrolateral and dorsolateral) and the temporal lobe have been proposed to be important for WM^2^. Other cortical regions may also be involved in modal-specific forms of WM traces. For example, the posterior parietal cortex, Broca’s area and premotor/supplementary motor areas have all been implicated in speech-related WM mechanisms^3^. Studies indicate that certain characteristics of WM are, to some extent, genetically transmitted, while it is also known that WM performance reduces with age^4^.

Our understanding of WM mechanisms has been shaped by case studies of patients with either congenital or acquired neurological/speech conditions^5^. It has been suggested that the rare trait of backward-speaking is linked to WM ability^6^. This trait is described as an ability to spontaneously and accurately reverse words. Two strategies of word reversal were reported: (1) reversal according to the phonetic structure of the words (speech sounds) or (2) reversal according to their spelling (letters). Previous studies concerning backward-speech focused on lexical aspects of the trait^6^,^7^,^8^,^9^,^10^,^11^,^12^. Cowan *et al.* described the ability of two children to speak backwards and, in a follow-up study published several years later, they documented only a slight deterioration in backward-speech ability^8^,^9^. The authors also described an adult with the same trait^8^,^10^. Furthermore, they proposed that phonetically-guided backward-speakers have a unique “set of analytical procedures that map an auditory image of a word into a string of phonological units put into working memory”^6^. This hypothesis was supported by the fact that nonsense words and foreign words could also be reversed with ease. However, even though the data disfavored the probability of transferring lexical representations from long-term memory to WM, the role of long-term memory in the process of reversal was not fully ruled out. Around the same time, Cocchi *et al.* reported the trait in an Italian-speaking patient that gained this ability after neurosurgery at the age of nine^7^. The authors hypothesised that some neural inhibitory mechanisms might have been lost due to the operation, although no concrete evidence to support this was presented. Similarly, Jokel and Conn reported a case of backward-speaking in a patient who suffered a head injury which resulted in what appeared to be a conversion disorder^12^. Strikingly, the patient also gained the ability to mirror-write and -read. Additionally, a single study suggested that backward-speech might occur in conjunction with schizophrenia, post-seizure state, as well as the aforementioned conversion disorder^11^. None of the previous cases reported a familial occurrence of the trait.

In this report, we describe individuals from a Serbian family who present with the ability to speak backwards voluntarily. The father (59 years of age) and one of the daughters (26 years of age; hereinafter referred as the “proband”) self-report that they developed this ability at a young age, while the mother, the other daughter and all other living relatives do not possess the trait (Fig. 1A). In the father and proband, backward-speaking is guided by the phonetic structure, and reversal of sentences is characterised by preserved order of the words, while the phonemes (speech-sounds) of the individual words are reversed (as heard in Audio files 1,2,3,4,5–6). Both the father and the proband deny explicitly learning or practicing speaking backward and also state that no particular conscious method for word reversal is being used. The father self-reports that his ability to reverse phonemes has slightly reduced with age, which may be due to an age-dependent reduction in cognitive functions. The subjects do not exhibit any clinical features of reading, speech or neurological disorders.

We applied several approaches to investigate the neurological basis of the given ability in the proband. Moreover, given the presence of two family members with a shared characteristic, the apparent ease with which these individuals developed the trait, and previously reported genetic contributions to WM, we postulated that genetic changes may facilitate this ability. Thus, we investigated coding sequence changes through exome sequencing in six family members and copy number variations (CNV) using SNP array data from these individuals.

## Results

### Characterisation of backward-speech

In order to define the phenotype, we tested the ability of the father and the proband to reverse individual words and sentences and to speak backwards spontaneously. Both the father and proband were able to reverse either single words or sentences (Audio files 1,2,3–4) and demonstrated the ability to spontaneously speak backwards (Audio files 5 and 6). The father and the proband reversed individual words according to their phonetic structure while the order of words in the sentence is preserved, as heard in Audio files 3,4,5–6. The accuracy of the father’s skill to reverse words was reduced in comparison to the proband’s but it was still greater than that expected by chance (Table S2). Additionally, we observed that the proband performed the tasks with noticeably more ease than the father. The International Phonetic Alphabet (IPA) transcripts of the audio files can be found in Supplementary documents (Table S2, Table S3 and Table S4).

### Working memory supports backward-speech in the proband

**Figure 1:**
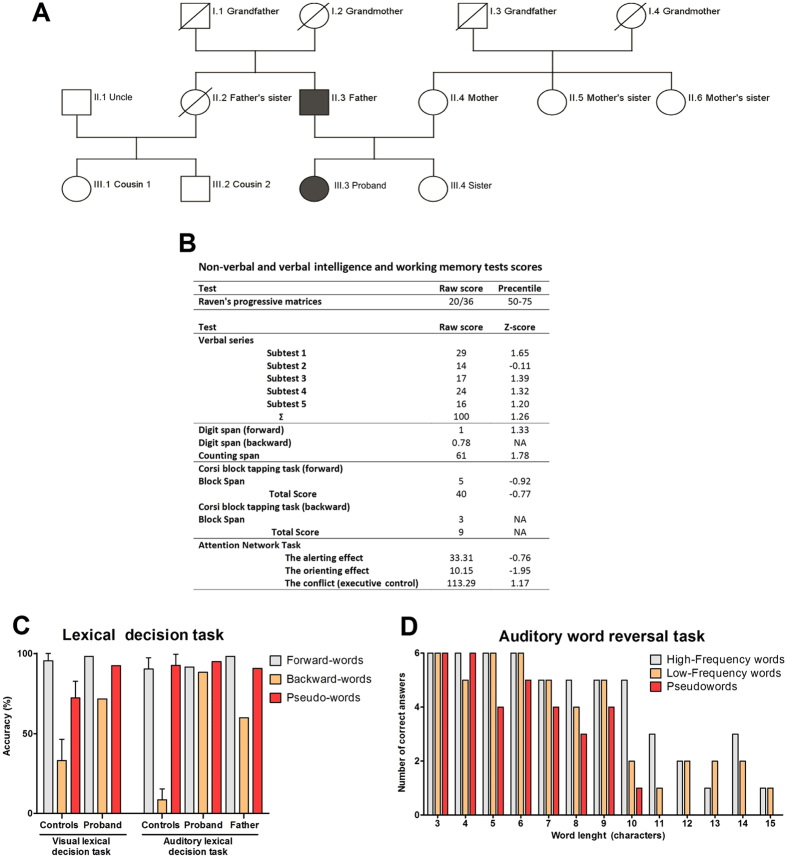
Pedigree of the family, behavioural test scores of the proband, and the analysis of the backward-speech trait. (**A**) Pedigree of backward-speaking family. Six individuals were sequenced-II.3- Father, III.3- Daughter 1 (proband), III.4- Daughter 2, III.1- Cousin 1, III.2- Cousin 2 and II.4–Mother. Individuals with the ability to speak backwards (father (II.3) and proband (III.3)) are shaded. Other relatives do not have this ability. (**B**) Scores of Raven’s progressive matrices, verbal intelligence tests and attention network task. (**C**) Lexical decision task accuracy of the control population, proband and father (median±IQR); (**D**) Number of correct answers in auditory word reversal task depicts the ability of the proband to reverse words.

We further examined the ability of backward-speech in the proband using standard Serbian psycholinguistic/behavioural tests. The proband scored high on tests of verbal intelligence (Fig. 1B). The results place her in the top 90th percentile of the Serbian population, with a verbal IQ score of 119 (+1.26 Standard Deviations (SD))^13^. Her performance was also above average in tests of digit and counting span but below average (−0.77 to −0.92 SD) on tests of visual-spatial working memory (Fig. 1B). Non-verbal intelligence was within the normal range (50_th_ − 75_th_ percentile) (Fig. 1B). In tests of attention, she performed below the population average (−10.76 and −1.95 SD, respectively) in alerting- and orienting-components, whereas her performance on the executive-component was above average (+1.17 SD) (Fig. 1B).

To assess the backward-speech in the proband, we performed an auditory word reversal task. The proband was able to reverse both real words and pseudo-words (i.e. words that were constructed to resemble the phonological structure of the real words presented in the experiment). Her performance was marginally more accurate for real words than pseudo-words and the accuracy of reversal decreased as word length increased (Fig. 1D). The proband’s performance was more accurate in re-reversing backward-words than normal forward-words (Compare Figure S1A with Figure S1B).

Auditory lexical decision tasks, in which the subject had to decide whether a word is a real word or a pseudo-word, were administered to both the father and the proband. Both individuals’ performances were highly accurate in distinguishing between forward-, backward- or pseudo-words, with performance that consistently exceeded control individuals (Fig. 1C).

Additional tests of auditory and visual lexical decision were completed by the proband and compared to a control group of randomly selected University students from Serbia. Lower accuracy was observed for the classification of backward-words in both auditory and visual tasks across both groups. The response latency of the proband for backward-words was significantly longer than that observed for forward-words but almost identical to that observed for the rejection of pseudo-words, thus indicating that no conscious strategy was employed for backward-words (Table S6). Logistic-regression modeling indicated that grapheme/phoneme sequence (letter and speech sound sequence) was the only factor that significantly impacted the accuracy of the proband’s performance, with forward-words being processed more accurately and faster than backward-words (Table S7). Grapheme/phoneme sequence by word frequency interaction was also found to affect the processing latency of the proband, with word frequency effect being significant only for forward-words. Analysis of errors revealed that, as expected, concrete nouns were significantly less error-prone than abstract nouns (Table S8). None of the other predictors reached significance, and there were no significant interactions. The effect of lemma frequency (where more common words are recognised faster), which is commonly observed in psychological experiments, was absent for backward-words. This indicates that the processing of forward- and backward-words differ and particularly suggests that backward-words do not have separate mental representations in the forms in which they appear. This is in accordance with the statement of the proband that she does not use any specific method for word reversal.

**Figure 2:**
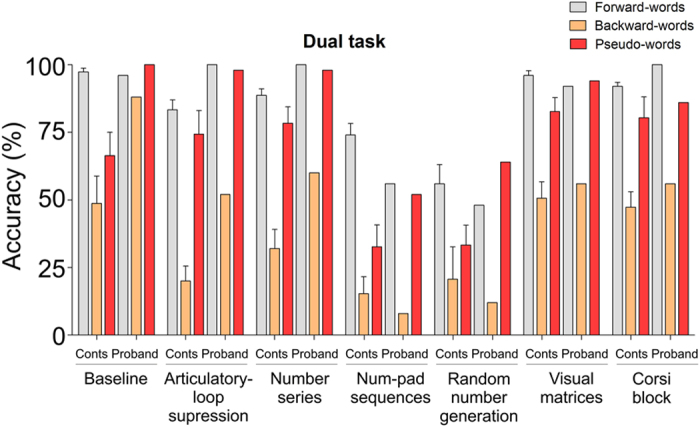
Dual task data directly implicate working memory in backward-speech. The accuracy data from dual task experiment is shown for the controls and proband (median±IQR) was visual lexical decision task while the concurrent tasks are written below the corresponding bars (detailed explanations of the concurrent tasks can be found in the Methods section).

The above data suggest that the proband employs WM strategies to reverse words. To investigate this hypothesis further, we employed a dual task experiment in the proband and additional control individuals (Fig. 2). In a visual lexical decision task at baseline, no significant differences were observed for the proband between tasks involving forward-, backward- and pseudo-words. This contrasts with controls, whose performance dropped significantly across both pseudo- and backward-words (Fig. 2, Table S9 and Table S10). However, when a concurrent task which loaded on WM was introduced, the proband’s accuracy in recognising backward-words dropped significantly and was in concordance with that observed in controls, while, in the case of forward- and pseudo-words, accuracy was comparable to that in the baseline condition (Fig. 1C). The effect of a concurrent task upon the recognition of backward-words strongly suggests that the subject utilises WM while performing an on-line reversal of words. In line with this, the proband also performed exceptionally well on the Stroop and Sternberg task (both for forward- and backward-conditions), supporting the observation that she has high capacity/performance of WM (Figure S2).

### Comprehension of backward-words is characterised by changes in neural activity associated with frontal and temporal brain areas in the proband

**Figure 3:**
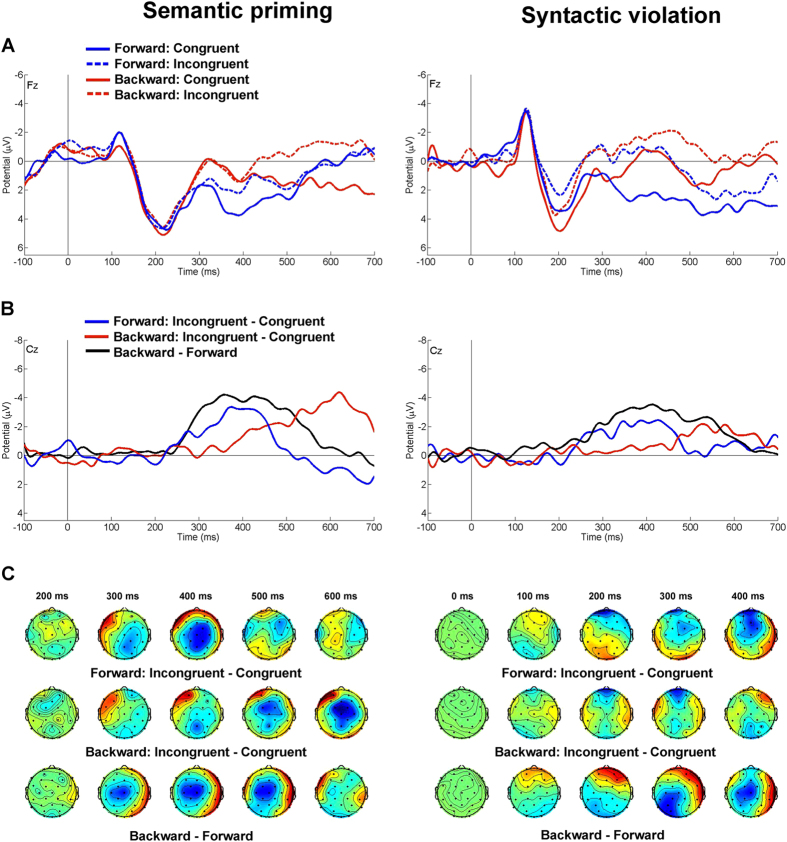
Word reversal is characterised by frontal negativity prior to congruence effect. **(A**) Event-related potentials obtained in the semantic (left) and syntactic (right) tasks for forward/backward-written, congruent/incongruent words, recorded at electrode Fz. A prominent congruence effect can be observed in the syntactic violation task only (solid vs. dashed waveforms), starting as early as 150 ms post-stimulus for both forward- (blue waveforms) and backward-written (red waveforms) words. This effect comprises of reduced amplitudes of the positivity peaking at 200 ms for syntactically incongruent words, yielding a negative incongruent vs. congruent difference around 200 ms at frontal midline electrodes in the scalp maps (**C**), irrespective of the direction of writing. Importantly, the effect of word reversal can also be observed in this time period at frontal electrodes, since backward-written words elicit larger ERP amplitudes at frontal electrode sites, resulting in a positive backward vs. forward amplitude difference in the scalp maps (**C**). (**B**) Incongruent vs. congruent difference waveforms obtained in the semantic (left) and syntactic (right) tasks for forward- (blue waveforms) and backward-written (red waveforms) stimuli, and the effect of word reversal (all forward-written vs. all backward-written words; black waveform) recorded at electrode Cz. In both tasks, the incongruence effect for forward words peaks around 375 ms. In the same time period (at 360 ms), an effect of word reversal can be observed, preceding the congruence effect for backward words, which is delayed by 245 ms. (**C**) Scalp maps for congruent vs. incongruent forward (upper row) and backward (middle row) words. In the semantic priming task (shown in the left), the congruence effect (central negativity) is substantially delayed for backward words, whereas it appears in the same time period in the syntactic violation task (frontal negativity). Crucially, a reversal effect (central negativity in the lower row) precedes the congruence effect of the backward condition in the semantic priming task only.

We next utilised electroencephalography (EEG) to examine the processing of backward-words in the proband. In the semantic priming task (in which the subject had to indicate whether a written word matches an image displayed beforehand), all word stimuli evoked the expected response - a prominent central negativity around 400–500 ms post-stimulus, which is known as the N400 (Figure S3A)^14^. As anticipated, this waveform was greater in amplitude for semantically incongruent words in both the forward- and backward-conditions. Additionally, backward-words evoked greater amplitude of N400 than forward-words. The incongruent-congruent difference waveforms at Cz (which represent the N400 amplitude difference measured for the incongruent vs. congruent conditions; Fig. 3B) revealed an earlier congruency effect for forward-words, with a similar effect peaking about 200 ms later for backward-stimuli (Fig. 3C). A very similar pattern was observed for the four conditions in a syntactic violation task (Fig. 3B, Figure S3A), albeit these effects were smaller. Crucially, a clear effect of word reversal, as indicated by the backward-forward difference waveform, appeared in both tasks around 400 ms (Fig. 3B), preceding the congruence effect in the backward-condition.

Syntactic violation produced a frontal negative difference at similar latencies (starting around 200 ms) for both forward- and backward-written words (Fig. 3A). This negative difference is reminiscent of the early left anterior negativity (ELAN), which is typically evoked by linguistic stimuli that violate word-category or phrase structure rules, but here it is restricted to midline electrodes (Fig. 3C)^15^. Given that this effect was not observed in the semantic priming task (Fig. 3A), it might be specific to the violation of syntax and not to semantics. Interestingly, the effect of syntactic violation at Fz was accompanied by an effect of reversal (Fig. 3A); backward-words evoked a larger frontal positivity around 200 ms, irrespective of syntactic congruence. In the syntactic violation task, the P600 was absent in both forward- and backward-conditions. However, this might reflect the absence of ambiguity in sentences used in the syntactic violation task^16^.

In the Sternberg working memory task, the participant’s performance was marginally worse for backward-words than forward-words (median response latency: forward: 581 ms (Interquartile range (IQR) = 149.25) vs backward: 640 ms (IQR = 283.00)). This might be due to the enhanced frontal negativity recorded during the retention period for backward-words (Figure S3B), which might indicate neural processes associated with increased working memory load in this condition.

### Working memory-related areas are active during backward-word production in the proband

**Figure 4:**
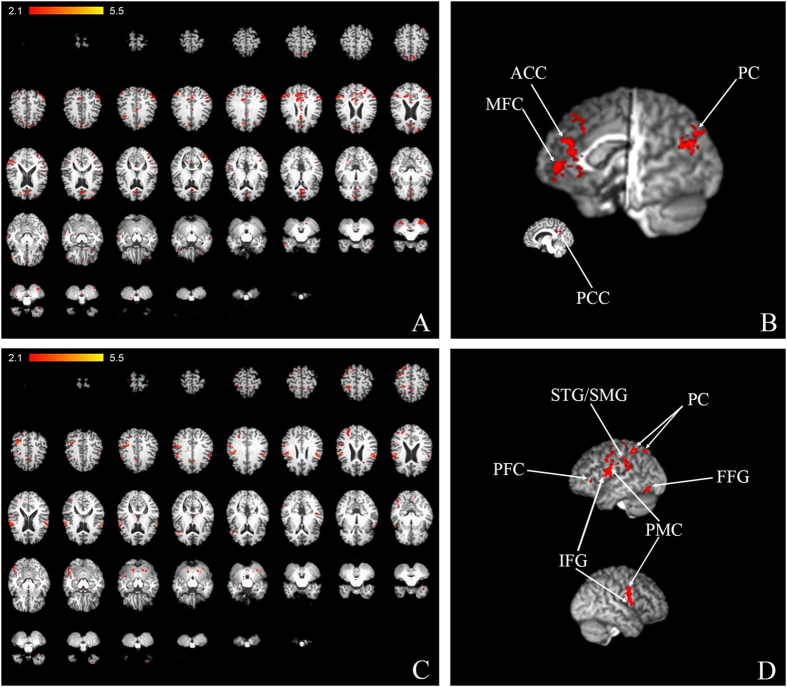
fMRI points to changes in activity of speech- and working memory-related areas. Maps of activity for normal-backward-speech in 2D (**A**) and 3D (**B**), and for backward-normal in 2D (**C**) and 3D (**D**): up-left hemisphere; down-right hemisphere. ACC–anterior cingulate cortex; PFC-prefrontal cortex PC-parietal cortex; PCC–posterior cingulate cortex; MFC–medial frontal cortex; SMG-supramarginal gyrus, STG-superior temporal gyrus, FFG -fusiform gyrus; IFG-inferior frontal gyrus; PMC-primary motor cortex.

In order to further examine the neural correlates of backward-“speech”, we utilised functional magnetic resonance imaging (fMRI) during tasks in which the proband mentally described her autobiography using forward- or backward-“speech”. Activation of the fusiform gyrus and prefrontal cortex of the left hemisphere and primary motor area of the right hemisphere was found to be exclusive for the backward-condition (Fig. 4C,D, Table S11, Table S12, Figure S8, Figure S9). Interestingly, there was a change in the pattern of activity within the parietal cortex when compared to forward-“speech” (Fig. 4B,D, Figure S8, Figure S9). During backward-“speech”, we observed higher activity of supramarginal gyrus, superior temporal gyrus of the left hemisphere, as well as of the inferior frontal gyrus and primary motor area of the left hemisphere compared to the forward-“speech” condition (Fig. 4C,D, Figure S8, Figure S9). The medial frontal cortex showed higher activity during forward-“speech” (Fig. 4A,B, Figure S8, Figure S9). The expected activation of the medial frontal cortex during narration of autobiography was observed both during forward- and backward-“speech” (Fig. 4A,B, Table S11, Figures S8, and S9). However, the activity was significantly higher during forward-“speech”^17^. Thus, it may be speculated that neural activity during backward-speech is centred on tasks related to the generation of non-standard words. Surprisingly, the right parietal cortex, anterior and posterior cingulate cortex, which are parts of the default mode network (DMN), were found to be statistically more active in forward-“speech”, indicating a higher extent of deactivation in backward-“speech”. This may be due to a degree of task-dependent deactivation of the DMN, or may point to a potential role for DMN in semantic processing, aligning with that already postulated^18^. The cerebellum was found to be activated in both forward- and backward-“speech”; however, no statistically significant difference in activity was observed (Table S11). This additionally points to an important cerebellar function in speech production and cognition which has been suggested by numerous reports^19^,^20^. The MNI coordinates (Montreal Neurological Institute and Hospital Coordinates) corresponding to areas in Fig. 3 can be found in the Supplementary tables (Table S12).

### Genetic variants may support backward-speaking

We employed exome sequencing to investigate the role of rare genetic variants in the observed trait. Six family members were sequenced (II.3- Father, III.3- Daughter 1 (proband), III.4- Daughter 2, III.1- Cousin (Paternal side of family) 1, III.2- Cousin (Paternal side of family) 2 and II.4–Mother, Fig. 1) generating 21,840Mb of sequence, of which 92% mapped to the genome providing 12.5% coverage. The average exome coverage across the six family members was 28.3 (range 11.8–38.7) and the median exome coverage was 74.8 (range 46.6–89.8). Seventy-five percent of targeted exome sequence had coverage >10-fold. Sequencing metrics can be found in Table S13. In total 784,444 variants were identified across all six individuals, of which 287,977 passed quality filters. Given the family structure and reported history, we hypothesised that the proband carried a coding mutation that conferred or contributed to her ability and that this mutation was inherited from her father. We therefore looked for rare coding variants of potential pathogenic significance that were passed from the father to the proband, but not to daughter 2 (i.e. a model of autosomal dominant inheritance, Fig. 1A). Our initial filter involved selecting those variants that were covered in at least five of the six exomes and were present in a heterozygous state in the father and the proband but not in any of the other four relatives sequenced (n = 3266). Of the 3266 shared variants, 505 were exonic. These variants were further filtered to remove all known sites of variation (from publically-available databases 1000 Genomes and NHLBI Exome Variant server, which together consist of data from 4769 individuals representing an expected allele frequency of 0.01%) and variants of unknown significance (synonymous or non-frameshift variants) leaving nine variants for Sanger sequencing validation. A flow diagram of the filtering process can be found in Supplementary figures (Figure S4).

Through Sanger sequencing, five variants (of which four were indel events) were found to represent false positives, three variants were confirmed to be present as heterozygous single base substitutions in the father (II.3) and the proband (III.3) but absent in all other family members (g.chr6:3105973, g.chr11:8161603, g.chr11:10874596;hg19), and the remaining one (g.chr15:28491062) was confirmed to be present in the proband (III.3) but not in any other family members (Table 1, Figure S5.). None of the validated variants were detected in 150 Serbian control samples. The three candidate variants were located in exon 8 of the *RIPK1* gene (c.C1264G, p.Q422E), exon 3 of the *ZBED5* gene (c.C1897T, p.R633C) and exon 2 of the *RIC3* gene (c.G262A, p.G88R in all 3 coding isoforms). Two of the three co-segregating variants were predicted to be deleterious to protein function by at least one annotation algorithm (Table 1 and Table S14).

SNP arrays allowed the additional characterisation of copy number variants in all 6 family members. All CNV events identified in the family were common (overlap ≥50% or with ≥20 supporting structural variants in the DGV) indicating that these events do not contribute to the trait.

Subsequently, we checked the interacting partner of these proteins and found that all three candidate genes appear to be related via ubiquitination proteins, specifically via ubiquitin C (UBC) (Figure S6). Both RIC3 and RIPK1 were predicted to be direct interacting partners of UBC (score = 0.479 and 0.999, respectively), whereas ZBED5 was found to be related to UBC via EPS15 and CDKN1B (which are interacting partners of UBC). Thus, we investigated whether the non-synonymous changes identified in the three candidate genes could alter predicted ubiquitination sites in the corresponding proteins. None of the amino acid changes directly affected or introduced a Lysine residue and no gross alteration of ubiquitination was predicted for the sequence changes (Table S15). Further investigation of the mutation burden across proteins reported to interact with UBC, again indicated that ubiquitination is unlikely to represent a general mechanism for this trait (Table S16).

The expression of the three identified candidate genes in the human brain was examined using microarray data of six individuals in the Allen Brain Atlas. *RIC3* was found to be expressed in the frontal lobe (3/6), cingulate gyrus (4/6), parietal lobe (5/6), white matter (5/6) and cerebral cortex (6/6) (Figure S7). *ZBED5* shows similar expression patterns to *RIC3*. However, it was also expressed in the ventral thalamus (6/6). The pattern of *RIPK1* was more distinct but showed some similarities to the expression of *UBC* (results not shown). *RIPK1* is expressed in several brain regions including amygdala (5/6), globus pallidus (6/6), claustrum (6/6) and ventral thalamus (6/6) (Figure S7).

## Discussion

In this article, we report results from behavioural testing and neuroimaging of a proband with an ability to speak backwards voluntarily and exome sequencing of individuals from her nuclear family. Our studies of the proband provide evidence that the on-line reversal of words is executed in the WM, although lexical identity may be accessed and could support reversal. Additionally, we were able to observe the activation of WM-related and visual-word-imagery-related brain areas in the proband during backward-speech. Furthermore, exome sequencing of six family members uncovered three rare exonic mutations in the *RIC3*, *RIPK1* and *ZBED5* genes that co-segregated with the trait in this family. Although the family structure and sample size cannot allow the definitive identification of contributory variants, in combination with the behavioural and neurological data, we suggest that variants in these genes may contribute to backward-speech and thus represent putative candidate genes for WM-related pathways.

In both the father and proband, the backward-speech trait is characterised by voluntary and rapid rearrangement of speech units, either guided by phonetic structure or word morphology^6^. Although both acquired and untaught backward-speech have previously been described, familial untaught backward-speech has not been documented before. In the early reports on backward-speakers, it was hypothesised that a representation of backward-words is deposited into WM and then scanned in reverse or else reordered in the WM. It was suggested that a set of analytical procedures exist to orchestrate the mapping of the mental image of a word into a string of phonemes to the WM while the lexical identity could be used to clarify the stimuli^6^. The importance of WM in various aspects of language-processing and language-learning has been supported by compelling evidence from children affected by specific language impairment (SLI), in whom a reduction in phonological storage capacity has been observed^21^, and in dyslexia, which has been described as a phonological processing difficulty^22^. Furthermore, genetic contributions to SLI and dyslexia have been suggested to involve variations in genes related to WM performance^23^.

Differences in the proband’s neural processing of backward-speech were demonstrated both by behavioural techniques and neuroimaging. The absence of a lemma frequency effect for backward-words pointed to a different mode of word processing between forward- and backward-words^24^. It is highly likely that word reversal is occurring on-line and that lexical identities of backward-words are not present. Furthermore, dual task performance of the proband, alongside the event-related potential (ERP) analysis of the Sternberg working memory task, suggests that WM is involved in the process of word reversal, as has been previously hypothesised^6^. The proband was able to reverse both normal words and pseudo-words, and the number of phonemes in the longest pseudo-word (ten phonemes) is in concordance with the capacity of WM (as seen in the digit span task), strengthening the conclusion that the WM is involved in this task. However, the long-term memory also appears to support the reversal process as the length of the longest real word that was successfully reversed exceeds that of the longest pseudo-word. Additionally, the proband is more successful in reversing words that are already backward, suggesting that she accesses long-term storage to find candidate words that ‘fit the pattern’. The error analysis supports this hypothesis and further indicates that candidates from the long-term memory are selected by similarity (e.g. harmony instead of harmonization).

Accordingly, processing (i.e. activity/hemodynamic) differences were also observed in the EEG and fMRI experiments. There are some mismatches between EEG and fMRI data, which may be explained by the different stimulation paradigms used (e.g. EEG tasks were mostly focused on word comprehension, whereas fMRI was focused on the production of backward-speech), or by the different nature and output measurement of the techniques. Notably, a unique ERP pattern related to backward-word recognition was observed across the EEG tasks. The EEG data indicate that semantic analysis of backward-written words is more demanding as indexed by an increase in N400 amplitude and the delay in N400 effect elicited by prime picture-target word incongruence. Crucially, we found a temporal dissociation between the effect of word reversal and semantic incongruence in the backward condition, indicating that the integration of word meaning into the context of the prime stimulus requires prior semantic analysis of backward-words. In the syntactic task, the effect of word reversal evoked modulation of early ERP amplitudes at frontal electrodes, without affecting the neural signatures of syntactic violation. Given that context-dependent semantic integration and the analysis of syntactic structure are associated with temporal and frontal lobe activity, respectively^15^, our ERP results point towards the involvement of these regions in backward-word comprehension. This is particularly interesting in light of a rising number of studies which suggest that frontal ERPs are related to high WM load^25^, as well as to encoding and early item storage. Recently, the basal forebrain, which is known to be major cholinergic output system, was suggested to be responsible for ERP generation in the frontal region^26^. It is known that cholinergic projections from the basal forebrain to frontal cortex exist and that both types of acetylcholine receptors, muscarinic and nicotinic, are expressed in the basal forebrain. Moreover, both types of acetylcholine receptors have been implicated in speech-related and WM operations^27^. Blockade of muscarinic acetylcholine receptors leads to a decrease in performance in language-related tasks, and a similar pattern of reading and spelling difficulties to that described in patients with Alzheimer disease^28^. Stimulation of α7 nicotinic acetylcholine receptors leads to sustained improvement of WM^29^ and acetylcholine receptors have been implicated in various cognitive disorders. Thus, it may be that changes within the cholinergic output system could modulate WM, increasing its performance/capacity and contributing to the reversal process in the proband. Interestingly, a study of families affected by Specific Language Impairment recently reported a significant overrepresentation of CNVs involving genes functioning in acetylcholine binding, including the α3, α7 and β4 nicotinic acetylcholine receptors, (GO:0042166, *CHRNA7*, *CHRNA3*, *ACHE* and *CHRNB4*) implicating a role for this pathway in speech and language disorders^30^.

Using fMRI imaging to investigate the proband’s ability to speak backwards, we observed a high hemodynamic response of areas that have been implicated in speech-related WM mechanisms^31^. The prefrontal cortex (verge of inferior frontal cortex), supramarginal gyrus, superior temporal gyrus, and parietal cortex have all been found to have a role in phonological processing and verbal WM^32^. These results thus support our conclusion that the phonological loop is important for backward-speech processing in the proband. Interestingly, the left fusiform gyrus was found to be metabolically active only during backward-speech. The interaction between orthography and vocal rendition in backward-speech has already been proposed before^8^ and multiple lines of evidence for the involvement of the left fusiform gyrus in visual semantics can be found in the literature. For example, hypo-metabolism of the fusiform gyrus has been found to occur in semantic dementia^33^, and higher activity of the fusiform gyrus was found to be related to visual mental imagery^34^. However, in blind people, the left fusiform gyrus was found to be active during listening, suggesting that it could act as a part of phonological processing network and that it also has a role in speech processing^35^,^36^. Moreover, it has been observed that this area is active during spelling, and, accordingly, a role in the retrieval of orthographic whole-word representations has been suggested^37^. It was also reported that the interplay of the prefrontal cortex and the fusiform gyrus exists in visual working memory-related mechanisms^38^. We speculate that as backward-speech is a highly demanding task, activation of the left fusiform gyrus could possibly support reversal, i.e. it may be that the proband uses an orthographic representation of words to facilitate the reversal process during backward-word production.

The interaction between orthography and vocal rendition of backward-speech has been previously reported for English-speaking people^8^. In contrast, the Serbian language has a shallow orthography compared to English. As the proband had a sub-average visual-spatial WM performance, we propose that the neurophysiology of backward-speech may differ between different languages and this would represent an interesting area for further investigations.

Taken together, based on the EEG and fMRI data, we propose that the cholinergic system might be involved in the modulation and mechanisms of WM, while areas, such as the fusiform gyrus, may possibly facilitate backward-words production in the proband.

As two family members share the trait, we hypothesised that inherited rare genetic changes might facilitate this ability. We therefore modelled the trait as a dominant effect facilitated by a rare variant with high penetrance, thereby allowing us to select putative variants of contributory effects. Within this model, we identified three novel exonic variants that co-segregate within the family and are predicted to be functionally relevant by at least one prediction algorithm (g.chr6:3105973, g.chr11:8161603, g.chr11:10874596). The genes carrying these variants (*RIC3*, *RIPK1* and *ZBED5*) are all expressed in brain and represent potential candidates that may be functionally relevant to WM. The *RIPK1* gene codes for serine-threonine kinase 1 involved in TNFR1 signaling, which promotes NF-κB activation and cell survival, apoptosis or necroptosis^39^. While *RIPK1* has a crucial role in neurodevelopment^40^, polymorphisms in *RIPK1* have been associated with cancer and immune disorders, and overexpression of *RIPK1* can be seen in several cancer types (data publically available at https://www.oncomine.com). Therefore, it is less likely that the variant found is functional. The *ZBED5* gene is a member of the Buster family of proteins with a variety of different functions^41^. However, not much is known regarding the specific role of this gene. The *RIC3* gene is of particular interest as a WM-related candidate gene, as it encodes a molecular chaperone of nicotinic acetylcholine receptors (nAChRs) and 5-hydroxytryptamine receptor (5-HT_3_R) with an evolutionarily conserved function^42^. RIC3 plays a key role in the maturation of neurotransmitter-gated ion channels, which, in turn, mediate fast synaptic excitation and modulate synaptic release^42^. In particular, this chaperone is required for efficient folding and assembly of homomeric α7-nicotinic acetylcholine receptors, providing a functional link to the EEG and fMRI data collected in this family. Overlapping expressions profiles in the human brain can be seen for *RIC3* and *CHRNA7*, the gene encoding the α7 subunit^43^. Interestingly, both genes were found to be expressed in brain regions related to various aspects of learning and memory. Although all three candidate proteins interact with UBC, this did not reach significance (raw P = 0.09). Since over 8000 proteins have been described to interact with UBC (http://hintdb.hgc.jp/htp/proteins/P0CG48.html), and no increase in mutation burden in UBC-interacting proteins could be identified, it seems unlikely that this function is responsible for the phenotype observed.

In this study, we identified a non-synonymous variant in *RIC3* gene causing the substitution of the glycine 88 with an arginine. This residue is located within a poly-glycine stretch, found inside the proline-rich linker region. Studies of this protein region have found that the complete deletion of the linker leads to an inability to promote α7-nAChR surface expression. This effect could be restored by different small portions of the region^44^, suggesting that a number of specific residues of the proline-rich region are necessary for the functional activity of RIC3. Moreover, the capacity of RIC3 protein in the maturation of α7-nAChRs has been observed for different isoforms of *D. melanogaster RIC-3* containing exon 2: the presence of 40 additional residues in the linker region may also disrupt the function of this protein domain^45^. Therefore, it is possible that the amino acid change G88R may exert an effect on the protein activity that is relevant to the phenotype observed. Functional studies will be required to explicitly demonstrate this effect.

Several studies suggest a potential WM-related role of *RIC3*. Elevated *RIC3* expression has been detected in post-mortem brains of patients with schizophrenia or bipolar disorder, suggesting that dysregulation of RIC3 activity may be associated with cognitive impairment^46^. Strikingly, it was hypothesised in one of the early reports that backward-speech might be associated with schizophrenia^11^. Furthermore, *RIC3* was found to be associated with multiple sclerosis^47^ and cognitive maintenance^48^. Based on the literature and our findings, we speculate that expression of *RIC3* in the basal forebrain, WM areas, and/or fusiform gyrus could be important for WM-related and word processing-related mechanisms via alterations of α7-nAChR signalling in the brain and that this mechanism contributes to the backward-speech phenotype observed in this family.

Although the evidence provided in the study was obtained by multidisciplinary approach there were some unavoidable limitations: firstly, most of the behavioural tests and neuroimaging were done solely in the proband, due to practical constraints. Secondly, in the absence of age-matched backward-speakers, the proband served as her own control in EEG- and fMRI-related experiments, which were used to compare the processing of forward- and backward-words. Lastly, as for all case studies, the small sample size limits the conclusions that could be made from our genetic investigations; given the family structure, we would expect approximately 10% of the exome to be shared between the father and proband but not with other sequenced individuals (cousins, sister and mother). Our modelling of the trait as a highly penetrant dominant trait involving a rare functional variant allowed us to narrow our focus to three candidate variants within the co-segregating exome. Nonetheless, a single family will never enable the definitive identification of functional variants, and further case collections will be needed to validate the role of candidate genes in this trait.

In conclusion, single case studies have shaped neuroscience and, as demonstrated here, are still useful hypothesis generating tools. Our findings in a family with a peculiar phenotype point towards a specific relevant neural mechanism and new candidate genes and molecular pathways for working memory and speech-related memory tasks. The replication of our findings and the investigation of the identified candidate genes will contribute to our understanding of the biological underpinnings of speech and language and the neurological mechanisms which subserve this quintessential human trait.

## Methods

An overview of the tests performed can be found in Table S1.

### Ethics

All the segments of the study were performed in accordance with the ethical standards of the responsible committee on human experimentation and with the Declaration of Helsinki and were approved by the following committees; Ethical committee of the Department of Psychology, University of Novi Sad; Ethical committee of the Faculty of Medicine, University of Belgrade (29/X-6); Ethical Committee of the Oncology Institute of Vojvodina, Sremska Kamenica (1959–10/2011) and the United Ethical Review Committee for Research in Psychology, Hungary (EPKEB) (2013/2). Participants signed an informed consent from the Ethical committee of Department of Psychology, University of Novi Sad. Behavioural tests were performed in the proband and, where appropriate, in a control population and the father. Neuroimaging was performed solely in the proband. Exome sequencing and SNP array were performed for all the nuclear family members, two cousins (paternal side of the family) and 150 ethnically matched controls (Sanger sequencing validation only).

### Behavioural tests

Behavioural testing was performed at the University of Novi Sad, Serbia on three separate occasions. Measurements of laterality, verbal and non-verbal intelligence, working memory, language processing, dual task series, and auditory word reversal task were collected as described below. Two different control groups of randomly selected University students from Serbia (median age for both groups was 20) were used for the lexical-decision tasks (n = 24 randomly divided into two groups: visual lexical decision task n = 12 and auditory lexical decision task n = 12) and dual task series (n = 6).

The laterality of the proband was determined with the Edinburgh Handedness Inventory^49^. The proband declared 100% preference for the use of right-hand.

Raven’s advanced progressive matrices (test time was 40 minutes) were used to determine a non-verbal intelligence score for the proband^50^. Verbal intelligence of the proband was assessed with a standardised test of verbal intelligence commonly used for Serbian speaking population. The test comprises of five sub-tests evaluating logical memory; concept fitting; proverb understanding; reasoning by analogy; and complying in a task formulated within a scrambled-word sentence^13^.

Computer-based versions of digit-span, counting-span, the Corsi block tapping task and the attention-network task (http://osdoc.cogsci.nl/) were used to evaluate working memory and attention in the proband^51^,^52^,^53^.

We tailored the auditory word reversal task to investigate the ability of the proband to reverse words and pseudo-words. The list of stimuli (Table S5) contained words and pseudo-words of increasing length (from 3 to 15 characters). All words were grouped into three categories: (1) high-frequency words, (2) low-frequency words and (3) pseudo-words (i.e. words that were constructed to resemble the phonological structure of the real words presented in the experiment). Half of the stimuli were regularly pronounced words while half were pronounced backwards. In total 78 combinations (13 × 3 × 2) with three words were used (high-frequency words were matched for word frequency across word length categories, as was the case with low-frequency words) generating 234 stimuli. The stimuli were presented orally and the proband had the task to reverse the order of characters. The response of the proband was recorded and transcripts were made (Table S5).

Auditory- and visual-lexical decision task experiments were conducted to investigate language processing of words with forward- and backward-sequence of phonemes in the proband, father (only auditory-lexical decision task) and a control population (n = 24 randomly divided into two groups: visual lexical decision task n = 12 and auditory lexical decision task n = 12). We selected 240 real-words and 240 pseudo-words and divided them into two experimental datasets. Within each dataset half of the words differed in frequency of use (high or low), denotation (concrete concept or an abstract concept), and phoneme sequence (forward or backward). This yielded 32 word groups, all of which were matched for word length in graphemes/phonemes and uniqueness point. Groups differing in concreteness were matched for word frequency, and *vice versa*. In the auditory-lexical decision task, participants were first presented with a fixation point which remained on the screen for 1500 ms, followed by a blank screen for 500 ms. Subsequently, an auditory stimulus was played via headphones. The auditory stimuli were spoken by a female native speaker who pronounced them embedded in a sentence. The stimuli were extracted using Praat software (http://www.fon.hum.uva.nl/praat/). In the visual-lexical decision task, the fixation point was presented for 1500 ms, after which the stimuli was presented and remained on the screen until a response was received (maximal time for response was set at 1500 ms). In both tasks, participants were instructed to respond by pressing the left mouse button if the stimulus was a real word (in a forward- or backward-form), and by pressing right mouse button if the stimulus was a pseudo-word (in either a forward- or backward-form). A practice session proceeded all experimental blocks. The stimuli used in the practice session did not appear later and were not included in the analysis. Accuracy/error rate and response latencies in lexical decision tasks were analysed in the rms package (http://cran.r-project.org/web/packages/rms/index.html) implemented in R statistical software (http://www.R-project.org).

The relevance of working memory components in the process of backward-word recognition was investigated in a series of dual tasks for both the proband and control group (n = 6). The participants had a main task of recognising visually-presented forward- and backward-words and a second concurrent task (explained below) that had to be performed simultaneously with the main task. Participants were instructed to accept as words any string of letters/phonemes that has a meaning when pronounced in either forward- or backward-order. The response was to be given by pressing the left button of the mouse for real words and right button of the mouse for pseudo-words. Prior to stimulus presentation, a blank screen was shown for 500 ms, followed by a fixation point for 1000 ms. The stimuli were presented for 400 ms, succeeded by a blank screen that remained until the response or the time-out of 2000 ms. We selected a total of 350 Serbian nouns of equal length (five letters/phonemes). These were divided into 14 datasets of 25 nouns that were matched for lemma frequency, based on the Frequency Dictionary of Contemporary Serbian Language^54^. Subsequently, we formed seven lists containing two randomly selected groups of nouns (a total of 50 nouns). Within each list, nouns belonging to one of the two groups were shown in reverse order of phonemes. Finally, to each list, we added 50 pseudo-words which differed across groups/lists. Additionally, we formed 20 practice trials that mirrored the main design but were not analysed. Concurrent tasks were selected to engage separate components of working memory: articulatory loop, phonological short-term store, spatial component, visual component (or cache), and central executive^1^ through the following tests: a) Lexical decision task: baseline condition; b) Articulatory-loop suppression: concurrent task was to repeat aloud a Serbian two-syllable word “de-da” (eng. *grandfather*), approximately once per second (natural tempo); c) Number series: concurrent task was to memorise a list of nine digits; d) Num-pad sequences: concurrent task was to press keys on a numerical pad in clock-wise direction, using left hand, without looking (participants were allowed to look in order to place fingers after losing track); e) Random number generation: concurrent task was to produce (pronounce aloud) a random sequence of digits; f) Visual matrices: concurrent task was to memorise a visually-presented 3 × 6 matrix of nine white and nine randomly positioned black squares; g) Corsi block: concurrent task was to memorise a series of five visually-presented blocks.

In addition, modified versions of the Sternberg working memory task and the Stroop task were completed by the proband at the Institute of Psychology, Faculty of Arts, University of Szeged, Hungary^55^. The Stroop task was completed to assess selective attention and processing speed^56^. In this task, the proband was shown forward- or backward-written colour names (‘blue’, ‘green’, ‘yellow’ and ‘red’) in Serbian or a string of letters ‘XXX’ serving as a control condition, and the proband had to indicate the colour of the writing. All stimuli were written in one of the four colours (blue, green, yellow or red), yielding five experimental conditions (forward-written-congruent colours, forward-written-incongruent colours, backward-written-congruent colours, backward-written-incongruent colours, and ‘XXX’ presented in all of the selected colours in equal frequency). Six blocks of 100 stimuli were presented. In each block, stimuli were presented in a random order. Response time and accuracy were measured.

A modified version of the Sternberg task was used to assess working memory. The proband was asked to memorise three visually-presented consecutive stimuli of either forward- or backward-written Serbian words or sinusoidal grating patterns^57^. Each stimulus was presented for 700 ms with a random delay period of 200–350 ms between images. After a retention period of 425–575 ms, the participant was shown a probe stimulus and asked to indicate by pressing a button if the probe matched either of the three study stimuli. Half of the trials required a ‘match’ response. Each of the three stimulus conditions consisted of ten blocks of 30 trials, with the blocks appearing in a pseudo-random manner. The EEG recording was performed and accuracy and response time were measured.

### Electroencephalography (EEG)

The electroencephalography (EEG) was performed on the proband at the Institute of Psychology, Faculty of Arts, University of Szeged, Hungary.

EEG was recorded with a 32-channel BioSemi ActiveTwo Amplifier (BioSemi B. V., Amsterdam, Netherlands), with the sampling rate of 1024 Hz. The Ag/AgCl electrodes placed in elastic caps were positioned in accordance with the extended international 10/20 system. The recording reference and the ground electrodes (Common Mode Sense and Driven Right Leg electrodes in the ActiveTwo System^58^) were placed in close proximity to the Cz position. Neural responses associated with semantic and syntactic-processing and related to the analysis of forward- and backward-written words were assessed in two tasks: the semantic priming task and syntactic violation task.

For the semantic priming task, 400 images of objects were selected from Snodgrass & Vanderwart picture set^59^. The pictures were shown for 250 ms and were followed by a forward- or backward-written noun. The proband had to indicate whether the meaning of the noun matched the image by pressing the appropriate button. The words remained on the screen until the response. Half of the nouns were congruent with the preceding image, whereas half were incongruent.

In the syntactic violation task, 400 sentences were presented. Each sentence consisted of three sections: a subject, a predicate and an object (e.g. “The grandmother washes the clothes.”). These three parts were presented consecutively with the last either being syntactically correct or incorrect (e.g. “The grandmother washes at the clothes.”). In half of the trials the last section was written backward. Therefore, 100 sentences were presented randomly in one of the four conditions (last word: forward-congruent, forward-incongruent, backward-congruent and backward-incongruent). In order to ensure that the proband read and interpreted the sentences, a probe question about the content of the actual sentence (e.g. “Was the grandmother washing the dishes?”) appeared randomly (for 20% of the sentences) and proband was asked to answer with “yes” or “no” by pressing one of the response buttons.

Continuous EEG data were analysed with the EEGLAB toolbox (http://sccn.ucsd.edu/eeglab/) for Matlab (MathWorks Inc., Natick, USA). Data were re-referenced to an average reference; epochs with 100 ms pre- and 700 ms post-stimulus intervals were extracted and visually inspected to remove those with ocular or other prominent artifacts (average rejection rate below 10%). In the semantic-priming task, only epochs with correct responses were included into the analysis. After applying a 30 Hz low-pass linear finite impulse response filter to averaged EEG data, event-related potentials (ERPs) were investigated at electrodes Fz and Cz. In addition, scalp topographies of each stimulus condition and topographies corresponding to differences between conditions were plotted for visual inspection.

### Functional magnetic resonance imaging (fMRI)

The functional magnetic resonance imaging (fMRI) was performed on the proband at Diagnostic Imaging Center, Oncology Institute of Vojvodina. The subject was scanned on a 3T MR Unit (Magnetom TRIO, Siemens, Erlangen, Germany). The functional images were obtained in the axial plane with the following parameters: field of view (FOV) 240 mm, slice thickness 3 mm, 36 slices per volume, matrix 64 × 64, flip angle 90°, repetition time (TR) 3 s and echo time (TE) 30 ms. Additionally, a high-resolution 3D T1-weighted sagittal scan was acquired (FOV = 256 × 256 mm, slice thickness 1.1 mm, 160 slices, voxel size 1 × 1 × 1.1 mm and acquisition matrix 256 × 256).

Imaging was performed in a block design (three blocks of altering active and rest state; 30 s each) to test spontaneous forward- and backward-“speech”, while rest was used as a control state. Before scanning, the subject rehearsed the tasks and was asked to remain still in the scanner with her eyes closed. During the active states of two independent recordings, the subject was asked to state her autobiography either normally or backward. In both tasks, she was instructed not to overtly speak but to “think of the words/sentences”.

Data were analysed using FSL (FMRIB’s software library; http://www.fmrib.ox.ac.uk/fsl). Pre-processing standard steps were applied: motion correction, non-brain removal and spatial smoothing by a Gaussian kernel of 4 mm^60^. To remove low-frequency artefacts, high temporal filtering was used (Gaussian-weighted least-squares straight line fitting, with sigma = 30.0 s). The general linear model as implemented in FSL was used for time series statistical analysis for each task and all images are non-linearly registered via the subject’s T1–weighted high-resolution structural images to the MNI 152 template^61^. The statistical images were initially corrected using a cluster threshold determined by Z > 2.3, and a corrected cluster significance of P = 0.05.

Higher-level statistics, i.e. analysis of differences between tasks, were carried out using a fixed effects model and statistical images were generated using clusters determined by Z > 2.1 at the corrected cluster significance threshold of P = 0.01.

### Exome sequencing

Genomic DNA for six family members (II.3- Father, III.3- Daughter 1 (proband), III.4- Daughter 2, III.1- Cousin (Paternal side of family) 1, III.2- Cousin (Paternal side of family) 2 and II.4–Mother, Fig. 1) was extracted from Oragene saliva collection kits (OG-500, DNA Genotek, Ottawa) using standard protocols. Exome capture was performed by the genomics facility at the Wellcome Trust centre for Human Genetics, Oxford, UK. Exomic DNA was captured from 1.5ug of genomic DNA using Nimblegen capture arrays (v3 exome, Roche Nimblegen, Basel, Switzerland), which provide an average target coverage of 80% of the exome at 20-fold across all samples. Samples were labelled, multiplexed and sequenced in a single lane on the Illumina HiSeq 2500 (Illumina Inc, San Diego, CA). Sequence reads were mapped to the human reference genome (Human37) using the BWA algorithm (http://bio-bwa.sourceforge.net/). Variants were called from BAM files with the Platypus algorithm (http://www.well.ox.ac.uk/platypus; v0.5.1) to produce a single combined VCF file.

All variants were annotated within ANNOVAR (http://www.openbioinformatics.org/annovar/). For each of the variants identified here, we present predictions from several algorithms. Although each of these are related, they all consider distinct aspects of functional prediction and can generate very different outcomes^62^. We, therefore, consider scores across algorithms rather than relying on a single algorithm to assess the functional significance of rare variants.

After filtering (see Figure S4 for a flow diagram of the exome filter process), all variants of potential significance were validated by Sanger sequencing in the family. Subsequently, 150 Serbian controls were screened for the validated variants. Primers for Sanger sequencing were designed in Primer3 and are available upon request.

In order to rule out the possibility of mosaicism of g.chr15:28491062 in the father, as the variant was identified by exome sequencing but not detected by Sanger sequencing, the paternal DNA was analysed with a colony PCR assay. A 430 bp PCR fragment was cloned into a pGEM-T vector (Promega) and transformed into XL1-Blue competent *Escherichia coli* cells. Colony PCR was performed on 175 individual transformants and products analysed by Sanger sequencing.

### CNV Analyses

In order to assess a putative role for copy number variants in the ability identified, we performed SNP genotyping and CNV analyses in all six family members available (II.3, III.3, II.4, III.1, III.2, III.4, Fig. 1A). All individuals were genotyped on the Illumina Infinium HumanOmniExpress-24v-1-0 beadchip, with the Infinium HTS protocol. Raw data were examined within the Illumina Genome Studio software. Putative copy number variants (CNVs) were identified using the algorithms PennCNV (http://penncnv.openbioinformatics.org/en/latest/; 2011 Jun16 version) and QuantiSNP (version 2.3)^63^. For both algorithms, CNV calls were made adjusting for GC content. Quality control (QC) was performed at both sample- and CNV-call- level. At the sample-level, in the PennCNV analysis all the individuals passed the QC, having an SD for the log R ratio (LRR) <0.35, a B allele frequency (BAF) drift value <0.002 and a waviness factor between -0.04 and 0.04; in the QuantiSNP analysis, all the individuals had an average SD for the LRR <0.3 or an SD for BAF <0.15. Predicted CNVs with <3 consecutive SNPs and a confidence value (PennCNV) or log Bayes Factor (QuantiSNP) of <10 were excluded. Only CNVs predicted by both QuantiSNP and PennCNV with an overlap of ≥50% were considered reliable and were reported according to the innermost boundaries (“consensus” coordinates, GRCh37/hg19) or, in cases where a CNV was split in different adjacent calls by one algorithm, the boundaries were merged according to full-length CNV call.

In addition to the individual-based PennCNV calling algorithm, members of the nuclear family (father, mother, daughter 1 and daughter 2) were also analysed using the quartet-based algorithm in PennCNV.

All identified CNVs were compared against the Database of Genomic Variants (DGV, UCSC genome browser hg19, Nov 2014). The CNVs were defined “rare” if they overlapped <50% with 20 or less Supporting Structural Variants in the DGV.

### Pathway analyses, Ubiquitin prediction and Burden Analysis

To investigate possible interacting partners of RIC3, RIPK1 and ZBED5 we used the STRING database (http://string-db.org/).

The effect of the non-synonymous candidate variants on the predicted ubiquitination profile was assessed using the predictor software UbPred (http://www.ubpred.org/). The predicted ubiquitination profile was compared between the Reference protein sequences (HERC2: NP_004658; RIPK1: NP_003795; RIC3: NP_078833, NP_001193600 and NP_001193601; ZBED5: NP_067034 and NP_001137139), with those made for the correspondent sequences carrying the amino acid change.

A list of proteins interacting with Ubiquitin C (UBC) was downloaded from HitPredict (http://hintdb.hgc.jp/htp/), a database of high-confidence protein-protein interactions. Among 8139 interactions, 5961 were predicted with a high-confidence score. We divided the family members into two groups, individuals presenting the trait (II.3 and III.3) and individuals without the trait (II.4, III.1, III.2, III.4), and we compared the burden of variants in proteins interacting with UBC by performing a T-Test. High-confidence and low-confidence interactions were considered separately.

### Gene Expression

The expression of all four candidate genes in the human brain was examined using microarray data in the Allen Brain Atlas (http://www.brain-map.org).

## Additional Information

**How to cite this article**: Prekovic, S. *et al.* Multidisciplinary investigation links backward-speech trait and working memory through genetic mutation. *Sci. Rep.* **6**, 20369; doi: 10.1038/srep20369 (2016).

## Supplementary Material

Supplementary Audio File 1

Supplementary Audio File 2

Supplementary Audio File 3

Supplementary Audio File 4

Supplementary Audio File 5

Supplementary Audio File 6

Supplementary Data

## Figures and Tables

**Table 1:** Variants of potential significance identified in family.

Gene	Chr	Start chr posn (hg19)	End chr posn (hg19)	Ref	Variant	transcript:exon:AAChange	freq^1^	PhyloP^3^	SIFT^4^	PolyPhen2 HDIV^5^
Validated										
*RIC3*	chr11	8161603	8161603	C	T	NM_001206671,NM_001206672, NM_024557:exon2:c.G262A:p.G88R	novel	2.625	0.03	0.999
*RIPK1*	chr6	3105973	3105973	C	G	NM_003804: exon8:c.C1264G:p.Q422E	novel	2.641	0.18	0.082
*ZBED5*	chr11	10874596	10874596	G	A	NM_001143667,NM_021211: exon3:c.C1897T:p.R633C	novel	2.274	0.01	1.000
*HERC2*	chr15	28491062	28491062	T	A	NM_004667: exon23:c.A3542T:p.D1181V	novel^2^	1.168	0.38	0.999
Not Validated										
*TMF1*	chr3	69096995	69097006	GTGAAGACTTGA	TTGGAGTCTTGC	NM_007114: exon2:c.850_861	novel			
*TMF1*	chr3	69097012	69097012	A	ACCTTAGGCTTT	NM_007114: exon2:c.844_844delins ACCTTAGGCTTT	novel			
*MFSD1*	chr3	158519825	158519825	C	CTGTCAAGACGGCG	NM_001167903,NM_022736: exon1:c.31_31delinsCTGTCAAGACGGCG	novel			
*PPP3CB*	chr10	75227315	75227315	T	A	NM_001142353,NM_001142354,NM_021132: exon9:c.A1104T:p.E368D	novel			
*C15orf40*	chr15	83677185	83677185	A	ACAAAATCTT	NM_001160113: exon3:c.481_481delinsAAGATTTTGT	novel			

1–control frequency across 1000Genomes (all samples) and exome variant server (all samples).

2–validated only in the proband.

Three functional predictions are shown here (PhyloP, SIFT and PolyPhen2). A larger set of predictions is presented in Supplementary table S14.

3–PhyloP considers conservation of given base at the DNA level across species. A positive score represents a conserved base.

4–SIFT considers the conservation of bases and motifs at the protein level. SIFT scores ≤0.05 are considered deleterious. 5 – Polyphen2 considers the physical properties of amino acids. The HumDiv algorithm (shown here) is most suitable for fully penetrant Mendelian mutations. Polyphen scores ≥0.95 are considered damaging.
