# Supplementary material for: Multidisciplinary investigation links backward-speech trait and working memory through genetic mutation
Source: Sci Rep. 2016 Feb 3;6:20369. doi: 10.1038/srep20369 (PMC4738289; doi:10.1038/srep20369)
Supplement: Supplementary Data [file srep20369-s7.pdf]

Multidisciplinary investigation links backward-speech trait and working memory through genetic mutation

Stefan Prekovic[a,*,#], Dusica Filipović Đurđević[b,c,#], Gábor Csifcsák[d], Olivera Sveljo[e,f], Oliver Stojković[g], Milica Janković[g], Katarina Koprivsek[e,h], Laura E Covill[i], Milos Lucić[e,f], Thomas Van den Broeck[a], Christine Helsen[a], Fabiola Ceroni[i], Frank Claessens[a] and Dianne F Newbury[i*]

[a]Department of Cellular and Molecular Medicine, Faculty of Medicine, KU Leuven, Leuven, Belgium

[b]Department of Psychology, Faculty of Philosophy, University of Novi Sad, Novi Sad, Serbia

[c]Laboratory for Experimental Psychology, Faculty of Philosophy, University of Belgrade, Belgrade, Serbia

[d]Institute of Psychology, Faculty of Arts, University of Szeged, Szeged, Hungary

[e]Diagnostic Imaging Center, Oncology Institute of Vojvodina, Sremska Kamenica, Serbia

[f]Faculty of Technical Sciences, University of Novi Sad, Novi Sad, Serbia

[g]Faculty of Medicine, University of Belgrade, Belgrade, Serbia

[h]Medical Faculty, University of Novi Sad, Novi Sad, Serbia

[i]Wellcome Trust Center for Human Genetics, University of Oxford, Oxford, UK. OX3 7BN

***corresponding authors**

[#]equal contribution

Corresponding author details:

Stefan Prekovic

Department of Molecular and Cellular Medicine

Faculty of Medicine

KU Leuven

Herestraat 49 bus 901

+32 1637 90 02

Belgium

3000 Leuven

stefan.prekovic@med.kuleuven.be

Dianne Newbury

Wellcome Trust Centre for Human Genetics

Roosevelt Drive

Headington

Oxford.

OX3 7BN.

+44 1865 287652

dianne@well.ox.ac.uk

Key words: speech, language, working memory, *RIC3*, *ZBED5*, *RIPK1*

SUPPLEMENTARY INFORMATION

Supplementary files accompanying this manuscript.

Audio files

Audio file 1 Audio file demonstrating the proband's reversal of words. The tester gives a word and the proband responds with the reversed word.

Audio file 2 Audio file demonstrating the father's reversal of words. The tester gives a word and the father responds with the reversed word.

Audio file 3 Audio file demonstrating the proband's reversal of sentences. The tester gives a sentence and the proband responds with the reversed sentence.

Audio file 4 Audio file demonstrating the father's reversal of sentences. The tester gives a sentence and the father responds with the reversed sentence

Audio file 5 Audio file demonstrating the proband's spontaneous backward-speech. The proband describes her favorite food.

Audio file 6 Audio file demonstrating the father's spontaneous backward-speech. The father describes his favorite food.

Figures

Figure S1. Auditory word reversal task: proband's performance for forward-words, backward-words, forward-pseudo-words and backward-pseudo-words.

Figure S2. Proband's scores for Stroop and Sternberg task. Accuracy is shown in percentages (%). Response time shown in milliseconds (ms). Values represent median+IQR.

Figure S3. EEG waveforms and scalp maps of the proband. A) Effects of congruence and reversal in the semantic priming and syntactic violation tasks at electrode Cz. The N400 waveform can be observed in both tasks and all conditions, being larger for backward- vs. forward- and incongruent- vs. congruent-stimuli. B) Backward- vs. forward-differential EEG activity during the retention period of the Sternberg task. Increased frontal negativity was recorded for backward-written words.

Figure S4. Flow diagram showing the filtering of the exome data.

Figure S5. Validation by Sanger sequencing.

Figure S6. UBC is a close interacting partner of RIC3 and RIPK1. And it might also be linked to ZBED5 via EPS15 and CDKN1B.

Figure S7. Expression data from Allen Brain Atlas of 6 subjects. FL - frontal lobe; Ins – insul; CG - cingulate gyrus; HF - hippocampal formation; PG - parahippocampal gyrus; OL - occipital

lobe; PL - parietal lobe; TL - temporal lobe; Amg – amygdala; BF - basal forebrain; GP - globus pallidus; Str – striatum; Cl – claustrum; ET – epithalamus; Hy – hypothalamus; SbT – subthalamus; DT - dorsal thalamus; VT- ventral thalamus; ME – mesencephalon; CC - cerebellar cortex; CN - cerebellar nuclei; BP - basal part of pons; PT - pontine tegmentum; MY – myelencephalon; WM - white matter; and SS - sulci & spaces.

Figure S8. fMRI maps of the proband for forward "speech". Spatial smoothing Full width at half maximum (FWHM)=4mm, corrected cluster threshold Z>2.3, corrected cluster significance P=0.05) registered on T1 high resolution subject brain images

Figure S9. fMRI maps of the proband for backward-"speech". Spatial smoothing Full width at half maximum (FWHM)=4mm, corrected cluster threshold Z>2.3, corrected cluster significance P=0.05) registered on T1 high resolution subject brain images**.**

Tables

Table S1. Details of all tests performed in the study.

Table S2. The proband and father were orally presented with 50 single word stimuli and instructed to reverse the word (as heard in Audio file 1 and 2, respectively). The errors are marked red and bold (proband: two errors; father: ten errors).

Table S3. The proband and father were orally presented with one sentence at the time and instructed to repeat the sentence backwards, in the way they usually do (as heard in Audio file 3 and 4 respectively). The instruction was kept neutral in order to capture the information on whether the order of words in the sentence was preserved or manipulated. The errors are marked red and bold (proband: one error; father: five errors).

Table S4. The proband and father were asked to talk about their favourite food while playing with language as they usually do. The errors are marked red and bold.

Table S5. Proband's response and stimuli used in auditory word reversal task

Due to size this table is provided as a stand-alone document.

Table S6. Lexical decision task reaction time of the proband and father.

Table S7. Error rates and processing latencies of the proband as observed in visual lexical decision task and auditory lexical decision task.

Table S8. Results of the analyses of errors and response latencies of the proband as observed in visual lexical decision task and auditory lexical decision task .

Table S9. Coefficients from logistic regression model of control group's (n=6) accuracy in Dual task.

Table S10. Coefficients from logistic regression model for the accuracy of the proband in Dual task.

Table S11. Proband: fMRI coordinates for areas active in forward- or backward-condition.

Table S12. Proband: fMRI coordinates for areas highly active only in forward- or backward-condition.

Table S13. Exome sequencing metrics

Table S14. Predicted effects of shared variants of putative significance that validated.

Table S15. UbPred Prediction

Table S16. Ubiquitination burden analysis

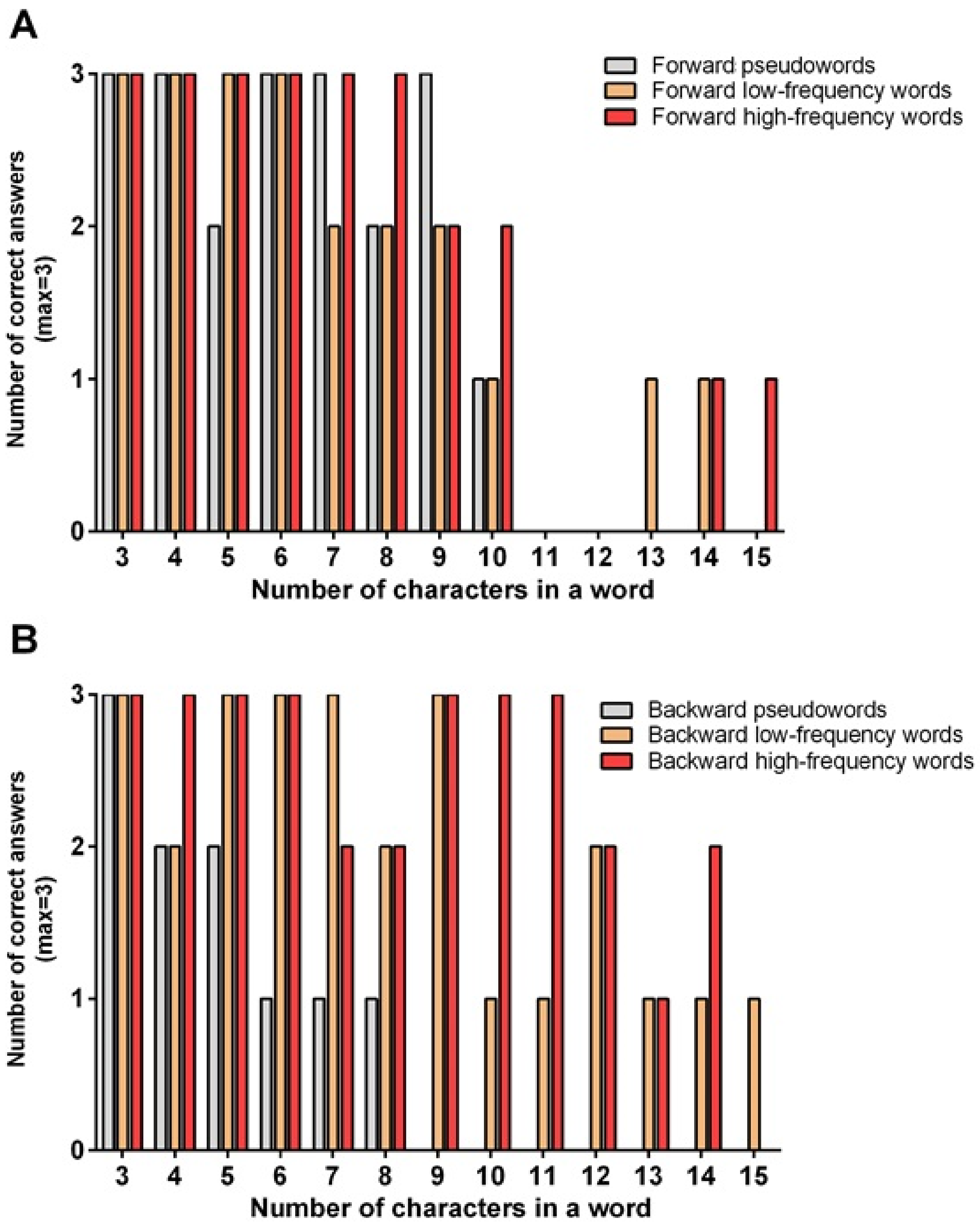

Figure S1. Auditory word reversal task: proband's performance for forward-words and backward-words.

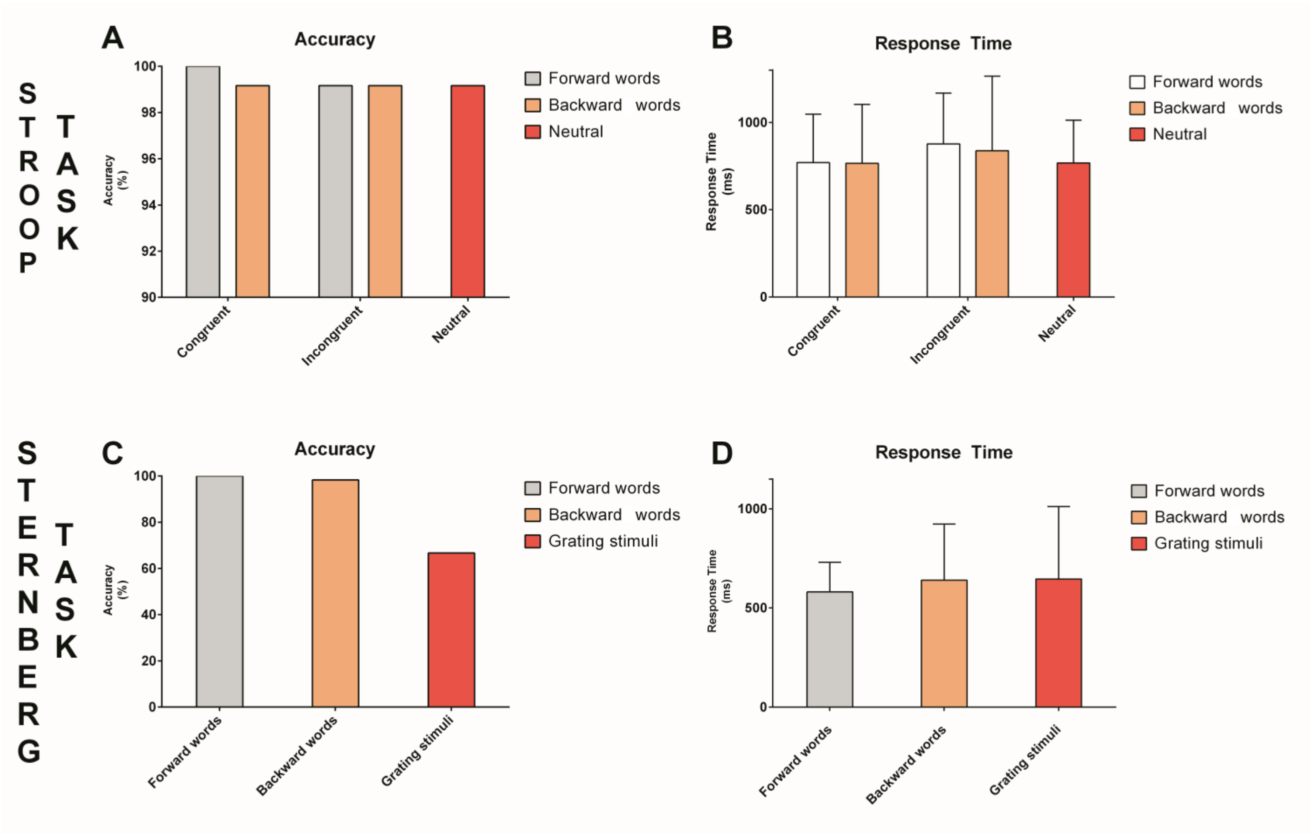

Figure S2. Proband's scores for Stroop and Sternberg task. Accuracy is shown in percentages (%). Response time shown in milliseconds (ms). Values represent median+IQR.

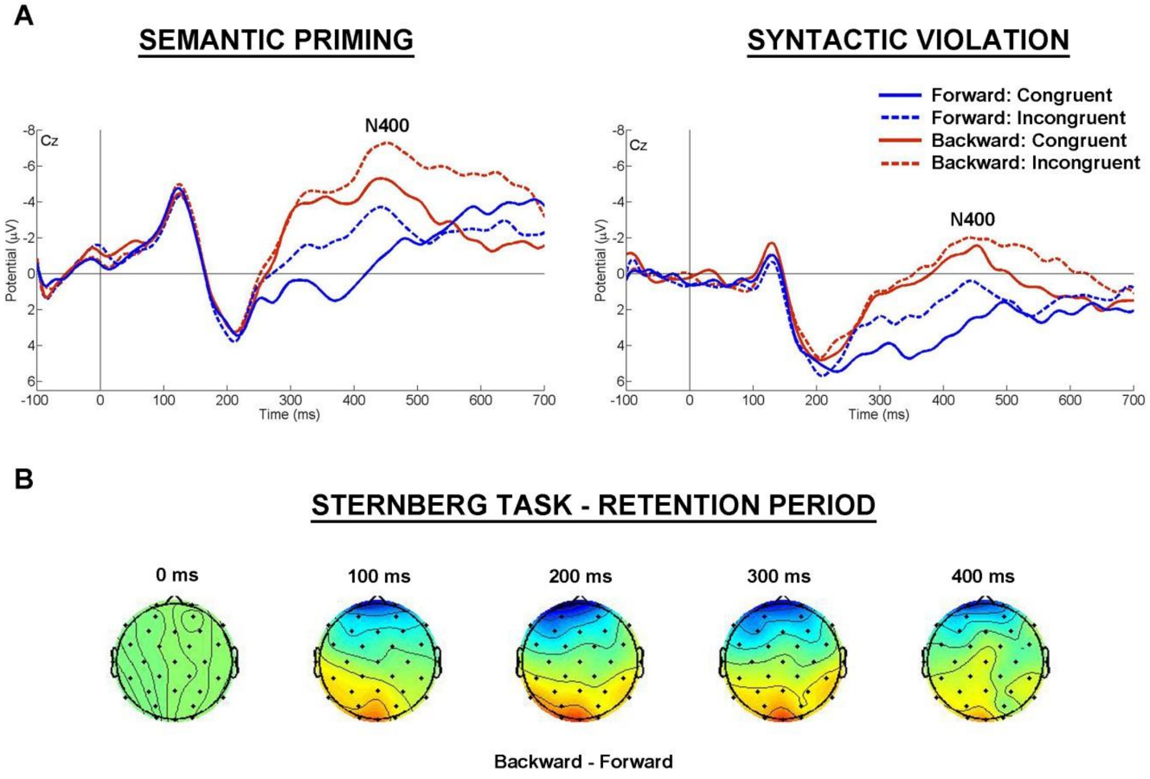

Figure S3. Event-related potentials and scalp maps of the proband. A) Effects of congruence (solid vs. dashed waveforms) and reversal (blue vs. red waveforms) in the semantic priming (left) and syntactic violation (right) tasks at electrode Cz. The N400 waveform can be observed in both tasks and all conditions, being larger for backward- vs. forward- and incongruent- vs. congruent-stimuli. B) During the retention period of the Sternberg task, the maintenance of backward-written words in WM was associated with an increased frontal negativity when compared to data obtained for forward-written words. This is evident from the scalp maps showing a prominent anterior negativity for the backward- vs. forward-difference waveform.

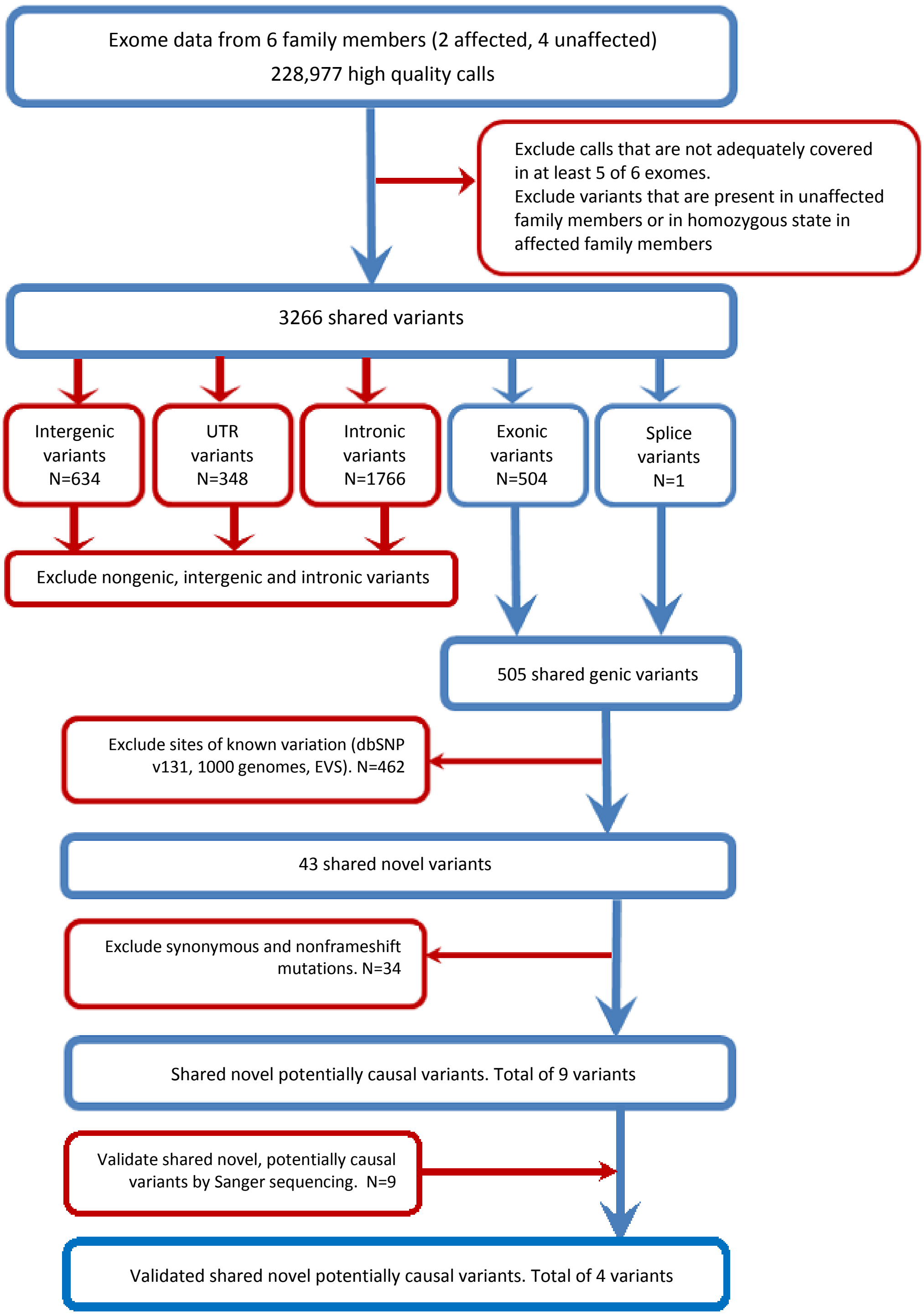

Figure S4. Flow diagram showing the filtering of the exome data.

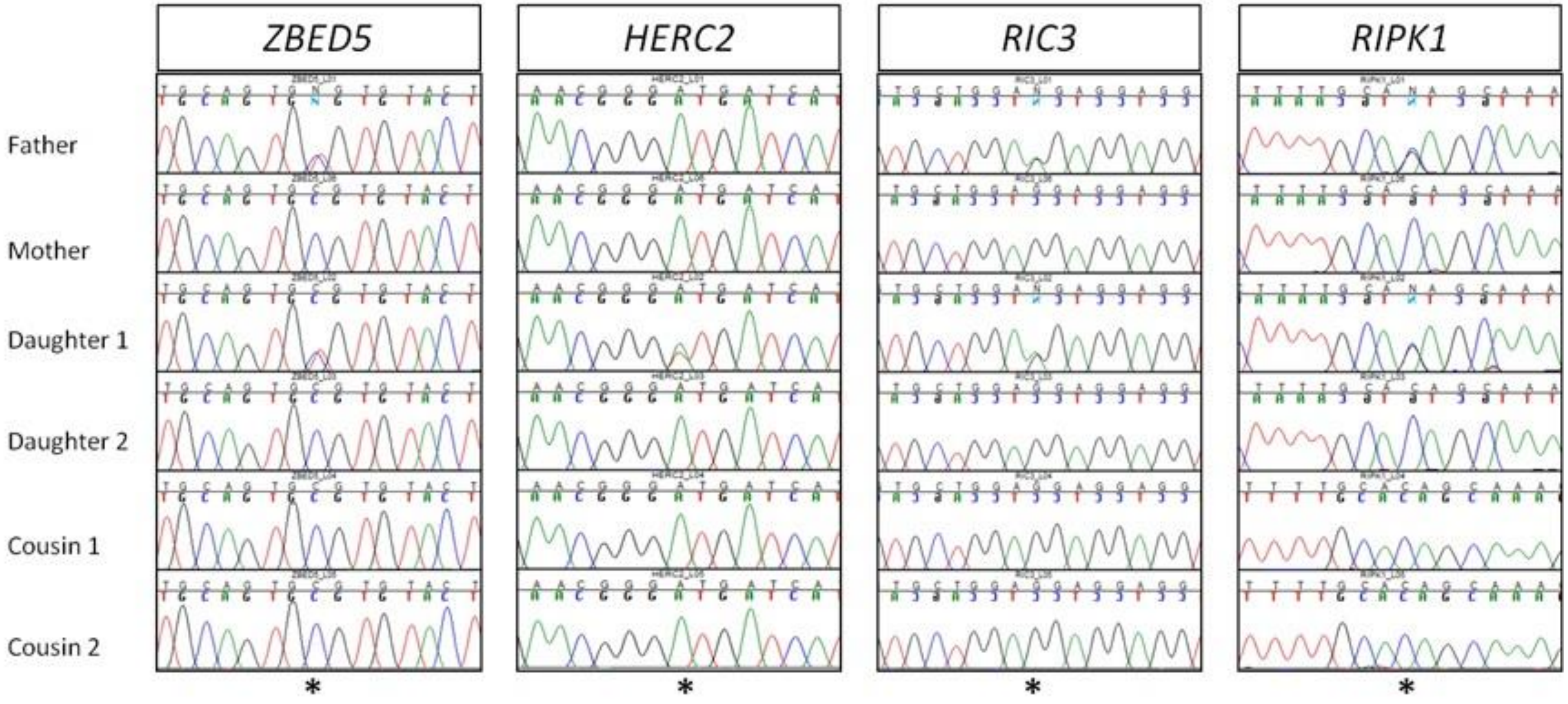

Figure S5. Validation by Sanger sequencing.

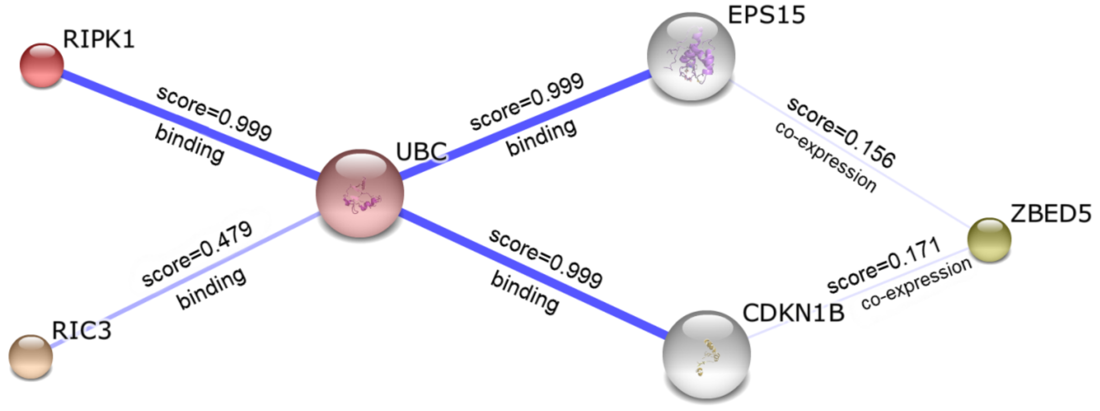

Figure S6. UBC is a close interacting partner of RIC3 and RIPK1. And it might also be linked to ZBED5 via EPS15 and CDKN1B.

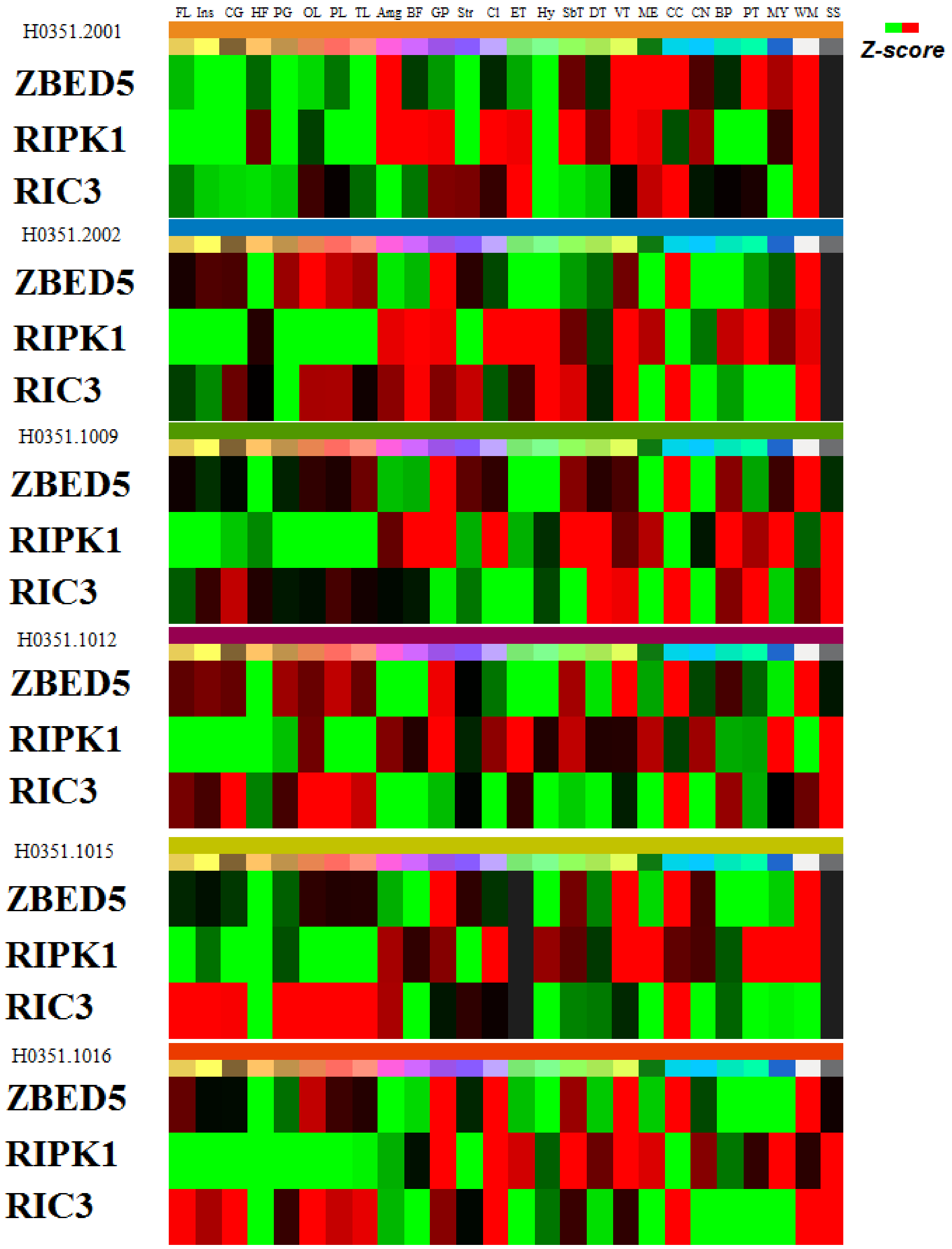

Figure S7. Expression data from Allen Brain Atlas of 6 subjects. FL - frontal lobe; Ins – insul; CG - cingulate gyrus; HF - hippocampal formation; PG - parahippocampal gyrus; OL - occipital lobe; PL - parietal lobe; TL - temporal lobe; Amg – amygdala; BF - basal forebrain; GP - globus pallidus; Str – striatum; Cl – claustrum; ET – epithalamus; Hy – hypothalamus; SbT – subthalamus; DT - dorsal thalamus; VT- ventral thalamus; ME – mesencephalon; CC - cerebellar cortex; CN - cerebellar nuclei; BP - basal part of pons; PT - pontine tegmentum; MY – myelencephalon; WM - white matter; and SS - sulci & spaces.

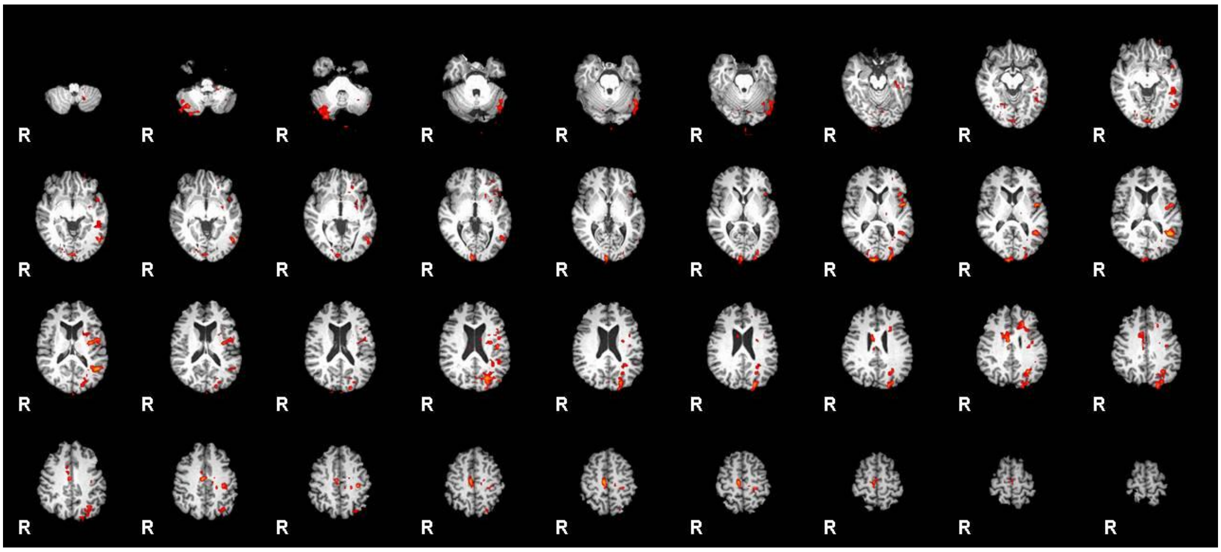

Figure S8. fMRI maps of the proband for forward "speech". Spatial smoothing Full width at half maximum (FWHM)=4mm, corrected cluster threshold Z>2.3, corrected cluster significance P=0.05) registered on T1 high resolution subject brain images**.**

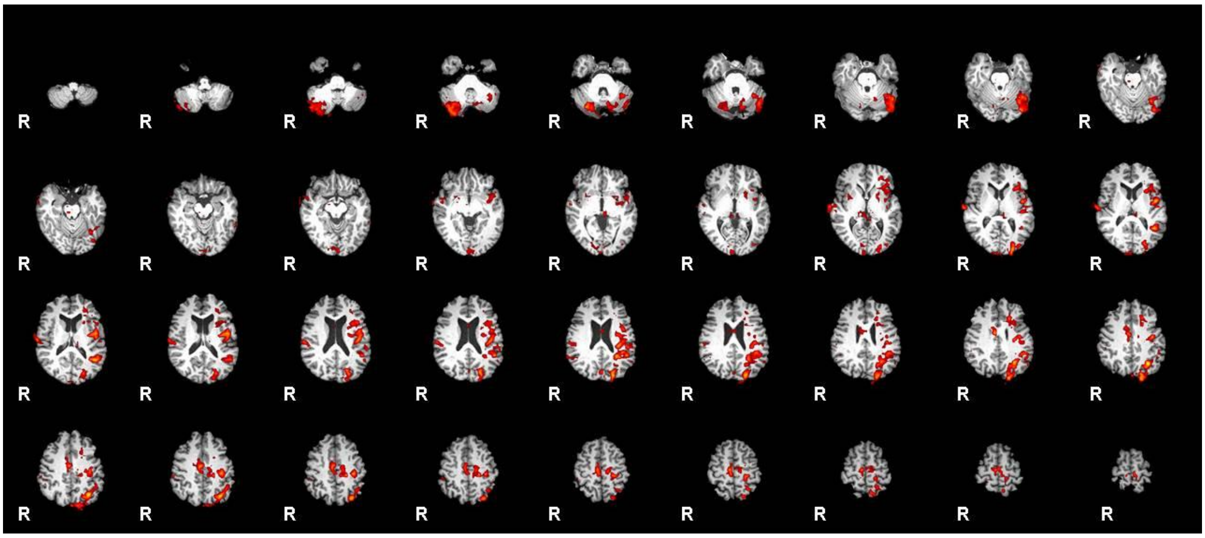

Figure S9. fMRI maps of the proband for backward-“speech”. Spatial smoothing Full width at half maximum (FWHM)=4mm, corrected cluster threshold Z>2.3, corrected cluster significance P=0.05) registered on T1 high resolution subject brain images **.**

Table S1. Details of all tests performed in the study.

Test	Subject(s)	Goal of the test
**Behavioral tasks**		
Word reversal	Proband<br>Father	To estimate performance in terms of accuracy.
Sentence reversal	Proband<br>Father	To assess whether the order of the words and/or the order of the phonemes is reversed.
Spontaneous backward-speech	Proband<br>Father	To get an insight into the trait.
Auditory word reversal task	Proband	To investigate the ability to reverse words and pseudo-words.
Edinburgh Handedness Inventory	Proband	To determine laterality.
Standardized test of verbal intelligence	Proband	To determine verbal intelligence score.
Standardized test of non-verbal intelligence	Proband	To assess non-verbal intelligence.
Digit-span	Proband	To describe WM capacity.
Counting-span	Proband	To describe WM capacity.
Corsi block tapping task	Proband	To assess visuo-spatial WM.
Attention network task	Proband	To asses individual components of attention.
Lexical decision tasks	Proband<br>Father<br>Controls (n=12 for each test)	To further estimate performance in terms of accuracy and speed and to test for the involvement of long-term memory in processing of backward-speech.
Dual task	Proband<br>Controls (n=6)	To investigate involvement of WM components in backward-speech.
Stroop task	Proband	To assess selective attention, processing speed and test for the involvement of long-term memory in processing of backward-words.
Sternberg task	Proband	To assess WM.
**EEG Tasks**		
Semantic priming task	Proband	To investigate neural signatures of the trait.
Syntactic violation task	Proband	To investigate neural signatures of the trait.
**fMRI Tasks**		
Spontantious 'speech'	Proband	To investigate neural signatures of the trait.
**Genomic Analysis**		
**Exome sequencing**	Proband<br>Father<br>Mother<br>Daughter 2<br>Cousin 1<br>Cousin 2<br>150 Serbian controls*	To investigate coding sequence changes.
**SNP array**	Proband<br>Father<br>Mother<br>Daughter 2<br>Cousin 1<br>Cousin 2	To investigate changes in copy number variations.

**used for Sanger sequencing validation only*

Table S2. The proband and father were orally presented with 50 single-word stimuli and instructed to reverse the word (as heard in Audio file 1 and 2, respectively). The errors are marked red and bold (proband: two errors; father: ten errors). IPA – international phonetic alphabet.

Item No.	Stimuli	Translation	IPA transcript	IPA transcript proband's response	IPA transcript father's response
1	pesma	song	/pesma/	/amsep/	/**asmep**/
2	vetar	wind	/vetar/	/ratev/	/**ratef**/
3	ulica	street	/ulitsa/	/aciluv/	/aciluv/
4	oblak	cloud	/oblak/	/kalbo/	/kalbo/
5	snaga	power	/snaga/	/agans/	/agans/
6	grana	branch	/grana/	/anarg/	/anarg/
7	senka	shadow	/senka/	/aknes/	/aknes/
8	jesen	autumn	/jesen/	/nesej/	/**nasej**/
9	cveće	flowers	/cvetɕe/	/etɕevts/	/**etɕevs**/
10	osmeh	smile	/osmex/	/xemso/	/xemso/
11	miris	smell	/miris/	/sirim/	/sirim/
12	metar	meter	/metar/	/ratem/	/ratem/
13	radost	joy	/radost/	/tsodar/	/ts..ts..tsodar/
14	prozor	window	/prozor/	/rozorp/	/rozorp/
15	tišina	silence	/tiʃina/	/aniʃit/	/aniʃit/
16	pogled	view	/pogled/	/delgop/	/delgop/
17	radnik	worker	/radnik/	/kindar/	/kindar/
18	daljina	distance	/daʎina/	/aniʎad/	/aniʎad/
19	plamen	flame	/plamen/	/nemalp/	/nemalp/
20	doktor	doctor	/doktor/	/rotkod/	/rotkod/
21	nedelja	Sunday	/nedeʎa/	/aʎeden/	/aʎeden/
22	pobeda	victory	/pobeda/	/adebop/	/adebop/
23	lepota	beauty	/lepota/	/atopel/	/atopel/
24	telefon	telephone	/telefon/	/nofelet/	/nofelet/
25	sloboda	freedom	/sloboda/	/adobols/	/adobols/
26	mladost	youth	/mladost/	/tsodalm/	/tsodalm/
27	planina	mountain	/planina/	/aninalp/	/aninalp/
28	predlog	suggestion	/predlog/	/golderp/	/golderp/
29	društvo	society	/druʃtvo/	/ovtʃurd/	/ovtʃurd/
30	partija	party	/partija/	/ajitrap/	/ajitrap/
31	problem	problem	/problem/	/melborp/	/melborp/
32	ministar	minister	/ministar/	/ratsinim/	/ratsinim/
33	svetlost	light	/svetlost/	/tsoltevs/	/tsoltevs/
34	politika	politics	/politika/	/akitilop/	/akitilop/
35	rezultat	result	/rezultat/	/tatluzer/	/tatluzer/
36	razgovor	conversation	/razgovor/	/rovogzar/	/…rovogzar/
37	trenutak	moment	/trenutak/	/**kanutert**/	/katunert/
38	preduzeće	company	/preduzetɕe/	/etɕezuderp/	/**atɕezuderp**/
39	skupština	parlament	/skupʃtina/	/anitʃpuks/	/anitʃpuks/

Item No.	Stimuli	Translation	IPA transcript	IPA transcript proband's response	IPA transcript father's response
40	detinjstvo	childhood	/detiɲstvo/	/otsɲited/	/**ovtsɲined**/
41	septembar	september	/septembar/	/rabmetpes/	/**rabmetes**/
42	republika	republic	/republika/	/akilbuper/	/akilbuper/
43	predsednik	president	/predsednik/	/**kindsenderp**/	/kindesderp/
44	delegacija	delegation	/delegacija/	/ajicageled/	/ajicageled/
45	proizvodnja	production	/proizvodɲa/	/aɲdovziorp/	/aɲdovziorp/
46	industrija	industry	/industrija/	/ajirtsudni/	/ajirtsudni/
47	teritorija	territory	/teritorija/	/ajirotiret/	/**ajirotoret**/
48	neprijatelj	enemy	/neprijateʎ/	/ʎetajirpen/	/**ʎatijerpan**/
49	obaveštenje	notification	/obaveʃteɲe/	/eɲetʃevabo/	/**eɲetʃvabo**/
50	prostorija	room	/prostorija/	/ajirotsorp/	/ajirotsorp/

Table S3. The proband and father were orally presented with one sentence at the time and instructed to repeat the sentence backwards, in the way they usually do (as heard in Audio file 3 and 4 Respectively). The instruction was kept neutral in order to capture information regarding word order. The errors are marked red and bold (proband: one error; father: five errors). IPA – international phonetic alphabet.

**Original sentence**	**Translation**	**IPA transcript**	**IPA transcript**<br><br>**proband's response**	**IPA transcript**<br><br>**father's response**
Pevaj!	Sing!	/pevaj/	/javep/	/javep/
Milica peva.	Milica sings.	/militsa peva/	/acilim avep/	/acilim avep/
Petar voli sladoxled.	Petar likes ice-cream.	/petar voli sladoled/	/ratep ilov delodals/	/ratep ilov delodals/
Jovana ide u školu pre podne.	Jovana goes to school in the morning.	/jovana ide u ʃkolu pre podne/	/anavoj edi u ulokʃ erp endop/	/anavoj edi u **uʃkol**… ulokʃ ulokʃ erp endop/
Kad padne mrak Zoran voli da ide na pecanje sa drugarima.	When it gets dark Zoran likes to go fishing with his friends.	/kad padne mrak zoran voli da ide na pecaɲe sa drugarima/	/dak endap karm naroz ilov ad edi an eɲacep as amiragurd/	/dak endap **kram** naroz ilov ad **na** eɲacep as amiragurd/
Samo onaj ko ima hrabrosti može da govori o trenucima kada se ponašao kukavički.	Only those who have courage are able to talk about moments when they behaved as cowards.	/samo onaj ko ima xrabrosti moʒe da govori o trenutsima kada se ponaʃao kukavitʃki/	/omas jano ok ami itsorbarx eʒom ad irovog o amitsunet adak es oaʃanop **ikvavitʃuk**/	/omas jano ok ami itsorbarx eʒom ad irovog **oak jano iok** es **oašanap** iktʃivakuk/

Table S4. The proband and father were asked to talk about their favourite food while playing with language as they usually do. The errors are marked red and bold. IPA – international phonetic alphabet.

Proband		
**IPA transcript**	**Serbian forward**	**Translation**
/aj mamen **aniʎemo** olej... milov ad **madej** atʃavs etatʃilzar iravts... andej do hijom hineʎimo alej **es** acipus **ivapeć** us **anzar** eɲipel... milov ad miʒart **etiltʃazer** etpecer okak hib amas ot ibes alivarpan i alivarps okat ad mamen **onterkok oniɲem** olej... ucip milov otaz otʃ ano eʒom okal ad es ivarpan i mamen iken inbesop suku et ote ʃab etʃuj mas alej uzoćirpak i olevikaz otaz iz ej ami **etiltʃalzer eliverp etiltʃalzer** esuku... milov as **amarkutʃep** as motalep as meʎavaktʃak i ote i ondej do **hionaʎimo** alej/	/ja nemam omiʎeno jelo... volim da jedem svaʃta, razlitʃite stvari... jedna od mojih omiʎenih jela su supica, ćevapi, su razne lepiɲe... volim da traʒim razlitʃite recepte kako bih sama to sebi napravila i spravila, tako da nemam konkretno omiʎeno jelo... picu volim zato ʃto ona moʒe lako da se napravi i nemam neki poseban ukus te, eto, jutʃe baʃ sam jela kaprićozu i ????? zato ????? ima razlitʃite prelive, razlitʃite ukuse, volim sa petʃurkama, sa pelatom, sa katʃkavaʎem i eto i jedno od omiʎenih jela/	I do not have a favourite dish... I like to eat all kinds of things, different things... one of my favourite types of food are soup, meatballs, are various bagels... I like to ask for various recipes in order to be able to make that for myself by myself, so I do not have some particular favourite food... I like pizza because it can easily be prepared and I do not have some particular favourite taste, for example, only yesterday I had capricciosa and ????? because ????? has various sauces, various tastes, i like it with mushrooms, with tomatos, with cheese and there you have one of my favourite types of food.

Father*		
**IPA transcript**	**Serbian forward**	**Translation**
/aj ogomn milov ʎusap...otaz im es adʑivs ogomn...(zato ʃto mi se mnogo svidʑa)... derop aʎusap milov i **aɲitegaj**... (jagɲetina, pored pasuʎa volim i jagɲetinu... je l' to to)... r... ʃer enetʃep... (reʃ petʃeno... ʃta bih joʃ voleo)...atʃ **bih** ʃoj **oleov**... kerub... derop **keruba** i azip... (ali)... ila ... kapi amen atʃ miloven... (ali nema ʃta ne volim)... ot ib olib **to ʃto**... to... ot otʃ ej...(najomiʎenije)... ejnoʎiman.../	/ja mnogo volim pasuʎ... zato mi se mnogo svidʑa... ...(zato ʃto mi se mnogo svidʑa)... pored pasuʎa voim i jagɲetinu (jagɲetina, pored pasuʎa volim i jagɲetinu... je l' to to)... reʃ petʃene (reʃ petʃeno... ʃta bih joʃ voleo)... ʃta bih joʃ voleo... burek... pored bureka i pizza... (ali)... ali... ipak nema ʃta ne volim... (ali nema ʃta ne volim)... to bi bilo to ʃto... to... to... ʃto je... (najomiʎenije)... najomiʎenije.../	I like beans very much... because I love it a lot... (because I love it a lot)... besides beans I like lamb...(lamb... besides beans I like lamn... is that it)... well done (well done... what else would I like)... what else would I like... cheese pie... besides cheese pie pizza, too... (but)... but... however there is not a thing that I do not like... (there is not a thing that I do not like)... those would be... those would be my... (favourite)... favourite...

* *Utterances that are pronounced in forward order of phonemes are put in the brackets; errors are underlined.*

Table S5. Proband’s response and stimuli used in auditory word reversal task

No of stimuli	Order in which stimuli were presented	Forwards or Backwards	Low/High-Frequency- or Pseudo-words	Word length	Lemma frequency	Stimulus	Correct response	Proband's response	Proband's response when she is not correct
1	63	backwards	pseudo	3	0	goc	cog	cog	
2	206	backwards	pseudo	3	0	dek	ked	ked	
3	172	backwards	pseudo	3	0	cur	ruc	ruc	
4	25	backwards	low-freq	3	1	ada	ada	ada	
5	223	backwards	low-freq	3	1	bob	bob	bob	
6	40	backwards	low-freq	3	1	išu	uši	uši	
7	78	backwards	high-freq	3	175	čam	mač	mač	
8	225	backwards	high-freq	3	179	ćom	moć	moć	
9	201	backwards	high-freq	3	201	rad	dar	dar	
10	42	backwards	pseudo	4	0	murt	trum	trum	
11	135	backwards	pseudo	4	0	hape	epah	epah	
12	51	backwards	pseudo	4	0	otil	lito	lito	
13	107	backwards	low-freq	4	1	morb	brom	brom	
14	83	backwards	low-freq	4	1	rate	etar	etar	
15	149	backwards	low-freq	4	1	ibol	lobi	ilob	ilob
16	122	backwards	high-freq	4	164	zalu	ulaz	ulaz	
17	220	backwards	high-freq	4	172	arok	kora	kora	
18	76	backwards	high-freq	4	180	agud	duga	duga	
19	12	backwards	pseudo	5	0	evaja	ajave	ajave	
20	126	backwards	pseudo	5	0	lazuh	huzal	hulaz	hulaz
21	148	backwards	pseudo	5	0	rejag	gajer	gajer	
22	85	backwards	low-freq	5	1	ilana	anali	anali	
23	146	backwards	low-freq	5	1	natub	butan	butan	
24	27	backwards	low-freq	5	1	remah	hamer	hamer	

No of stimuli	Order in which stimuli were presented	Forwards or Backwards	Low/High-Frequency- or Pseudo-words	Word length	Lemma frequency	Stimulus	Correct response	Proband's response	Proband's response when she is not correct
25	155	backwards	high-freq	5	176	canev	venac	venac	
26	54	backwards	high-freq	5	178	rakel	lekar	lekar	
27	6	backwards	high-freq	5	182	nadub	budan	budan	
28	56	backwards	pseudo	6	0	alenob	bonela	bolena	bolena
29	133	backwards	pseudo	6	0	azemaj	jameza	jameza	
30	1	backwards	pseudo	6	0	itikuv	vukiti	vukiti	
31	204	backwards	low-freq	6	1	aragon	nogara	nogara	
32	186	backwards	low-freq	6	1	derorp	prored	prored	
33	39	backwards	low-freq	6	1	sonoba	abonos	abonos	
34	102	backwards	high-freq	6	172	asevaz	zavesa	zavesa	
35	230	backwards	high-freq	6	179	itipuk	kupiti	kupiti	
36	72	backwards	high-freq	6	184	itisiv	visiti	visiti	
37	53	backwards	pseudo	7	0	itimero	oremiti	oremiti	
38	64	backwards	pseudo	7	0	itatrah	hartati	hartija	hartija
39	16	backwards	pseudo	7	0	azenamu	umaneza	umezan	umezan
40	15	backwards	low-freq	7	1	aciktap	patkica	patkica	
41	109	backwards	low-freq	7	1	zerorep	perorez	perorez	
42	13	backwards	low-freq	7	1	katsorp	prostak	prostak	
43	217	backwards	high-freq	7	174	sarečev	večeras	večeras	
44	197	backwards	high-freq	7	176	azevabo	obaveza	obaveza	
45	180	backwards	high-freq	7	186	itatsaz	zastati	zasititi	zasititi
46	147	backwards	pseudo	8	0	avonatfa	aftanova	natfa	natfa
47	34	backwards	pseudo	8	0	vitseloj	jolestiv	jolestiv	
48	43	backwards	pseudo	8	0	itiberha	ahrebiti	ahrebti	ahrebti
49	30	backwards	low-freq	8	1	nazicnok	koncizan	koncizan	
50	57	backwards	low-freq	8	1	naleunam	manuelan	munaela	munaela

No of stimuli	Order in which stimuli were presented	Forwards or Backwards	Low/High-Frequency- or Pseudo-words	Word length	Lemma frequency	Stimulus	Correct response	Proband's response	Proband's response when she is not correct
51	65	backwards	low-freq	8	1	sipovarp	pravopis	pravopis	
52	207	backwards	high-freq	8	172	tetilavk	kvalitet	kvalitet	
53	99	backwards	high-freq	8	174	itunerko	okrenuti	okrutni	okrutni
54	19	backwards	high-freq	8	188	vitkelok	kolektiv	kolektiv	
55	139	backwards	pseudo	9	0	itarelhod	dohlerati	dohlerati	
56	177	backwards	pseudo	9	0	notanabru	urbanaton	urbodan	urbodan
57	104	backwards	pseudo	9	0	ititsorku	ukrostiti	ukrsti	ukrsti
58	68	backwards	low-freq	9	1	cavadorad	darodavac	darodavac	
59	31	backwards	low-freq	9	1	ajisergid	digresija	digresija	
60	61	backwards	low-freq	9	1	nariludno	onduliran	onduliran	
61	189	backwards	high-freq	9	158	acindejaz	zajednica	zajednica	
62	55	backwards	high-freq	9	175	ititsorpo	oprostiti	oprostiti	
63	106	backwards	high-freq	9	220	rodasabma	ambasador	ambasador	
64	165	backwards	pseudo	10	0	ajinodiled	delidonija	delitioje	delitioje
65	159	backwards	pseudo	10	0	itilobokto	otkoboliti	oktoboliti	oktoboliti
66	226	backwards	pseudo	10	0	ititabjilb	blijbatiti	blivatiti	blivatiti
67	176	backwards	low-freq	10	1	itaviravaz	zavarivati	zavirivati	zavirivati
68	229	backwards	low-freq	10	1	onviseckus	sukcesivno	sukcesivan	sukcesivan
69	127	backwards	low-freq	10	1	itatevelko	oklevetati	oklevetati	
70	79	backwards	high-freq	10	83	rotizopmok	kompozitor	kompozitor	
71	87	backwards	high-freq	10	166	itidobolso	osloboditi	osloboditi	
72	185	backwards	high-freq	10	294	ajirotiret	teritorija	teritorija	
73	210	backwards	pseudo	11	0	intolamućen	nećumalotni	nećumolatni	nećumolatni
74	10	backwards	pseudo	11	0	iksdalgonet	tenogladski	tednologni	tednologni
75	95	backwards	pseudo	11	0	tedislebimu	umibelsidet	NEXT	NEXT
76	23	backwards	low-freq	11	1	mazirutnava	avanturizam	avanturizam	

No of stimuli	Order in which stimuli were presented	Forwards or Backwards	Low/High-Frequency- or Pseudo-words	Word length	Lemma frequency	Stimulus	Correct response	Proband's response	Proband's response when she is not correct
77	166	backwards	low-freq	11	1	ajicnevkole	elokvencija	eloknivecna	eloknivecna
78	190	backwards	low-freq	11	1	naralupopen	nepopularan	nepulopopen	nepulopopen
79	169	backwards	high-freq	11	85	ajicitsevni	investicija	investicija	
80	137	backwards	high-freq	11	91	ajicizopmok	kompozicija	kompozicija	
81	160	backwards	high-freq	11	393	indoranuđem	međunarodni	međunarodni	
82	84	backwards	pseudo	12	0	ajisnelepmog	gompelensija	NEXT	NEXT
83	154	backwards	pseudo	12	0	itapozimaklo	olkamizopati	olkamipozitpo	olkamipozitpo
84	9	backwards	pseudo	12	0	ovtstarpokli	ilkopratstvo	NEXT	NEXT
85	170	backwards	low-freq	12	1	itavartevorp	provetravati	provetravati	
86	216	backwards	low-freq	12	1	navitakovorp	provokativan	progovoriti	progovoriti
87	231	backwards	low-freq	12	1	narizinolbaš	šabloniziran	šabloniziran	
88	228	backwards	high-freq	12	26	ajicamitigel	legitimacija	legalizacija	legalizacija
89	158	backwards	high-freq	12	27	ajicazilibom	mobilizacija	mobilizacija	
90	82	backwards	high-freq	12	630	ajicnerefnok	konferencija	konferencija	
91	59	backwards	pseudo	13	0	iksnahirpubel	lebuprihanski	luberhist	luberhist
92	151	backwards	pseudo	13	0	nalitablezmog	gomzelbatilan	gomzbilbitnav	gomzbilbitnav
93	35	backwards	pseudo	13	0	tsonvarbonbal	labnobravnost	NEXT	NEXT
94	182	backwards	low-freq	13	1	ajicazinomrah	harmonizacija	harmonija	harmonija
95	222	backwards	low-freq	13	1	inoicutitsnok	konstitucioni	utisnuti	utisnuti
96	96	backwards	low-freq	13	1	navoziradnats	standarizovan	standarizovan	
97	75	backwards	high-freq	13	37	tsonvitkudorp	produktivnost	NEXT	NEXT
98	184	backwards	high-freq	13	39	navolpohudzav	vazduhoplovan	vazduhoplov	vazduhoplov
99	21	backwards	high-freq	13	49	navitavreznok	konzervativan	konzervativan	
100	46	backwards	pseudo	14	0	inkitsimajidoz	zodijamistikni	NEXT	NEXT
101	69	backwards	pseudo	14	0	ikčatekonramal	lamarnoketački	melanholičar	melanholičar
102	224	backwards	pseudo	14	0	ikčitsiradibag	gabidaristički	gabidistčiksi	gabidistikči

No of stimuli	Order in which stimuli were presented	Forwards or Backwards	Low/High-Frequency- or Pseudo-words	Word length	Lemma frequency	Stimulus	Correct response	Proband's response	Proband's response when she is not correct
103	209	backwards	low-freq	14	1	ajizarknisoidi	idiosinkrazija	idiosinkrazija	
104	98	backwards	low-freq	14	1	ajicnedopserok	korespodencija	korespondentan	korespondentan
105	92	backwards	low-freq	14	1	iksraketoilbib	bibliotekarski	biblioteka	biblioteka
106	196	backwards	high-freq	14	20	ikstetizrevinu	univerzitetski	univerzitetski	
107	11	backwards	high-freq	14	23	ajickurtsnoker	rekonstrukcija	rekonstrukcija	
108	193	backwards	high-freq	14	63	ikčamenondapaz	zapadnonemački	zapadnički	zapadnički
109	161	backwards	pseudo	15	0	acinčitokodezet	tezedokotičnica	dezertiran	dezertiran
110	152	backwards	pseudo	15	0	naritsiledgalag	galagdelistiran	galanitster	galanitster
111	48	backwards	pseudo	15	0	natamoimatlepmi	impeltamiomatan	implementirati	implementirati
112	203	backwards	low-freq	15	1	inoicaterpretni	interpretacioni	interpretacioni	
113	132	backwards	low-freq	15	1	cavitartsinimda	administrativac	administracija	administracija
114	47	backwards	low-freq	15	1	ovtsnajirategev	vegetarijanstvo	vegeterijanac	vegeterijanac
115	70	backwards	high-freq	15	12	ajicazilajiceps	specijalizacija	specijalan	specijalan
116	218	backwards	high-freq	15	14	etšilidargodorb	brodogradilište	brodograd	brodograd
117	128	backwards	high-freq	15	36	cinjšidogotesed	desetogodišnjica	deset..ne znam	deset… ("I don't know.")
118	37	forwards	pseudo	3	0	juk	kuj	kuj	
119	123	forwards	pseudo	3	0	gil	lig	lig	
120	212	forwards	pseudo	3	0	šad	daš	daš	
121	74	forwards	low-freq	3	1	čep	peč	peč	
122	211	forwards	low-freq	3	1	kub	buk	buk	
123	89	forwards	low-freq	3	1	ruž	žur	žur	
124	134	forwards	high-freq	3	174	sok	kos	kos	
125	62	forwards	high-freq	3	184	boj	job	job	
126	175	forwards	high-freq	3	192	laž	žal	žal	
127	93	forwards	pseudo	4	0	krza	azrk	azrk	

No of stimuli	Order in which stimuli were presented	Forwards or Backwards	Low/High-Frequency- or Pseudo-words	Word length	Lemma frequency	Stimulus	Correct response	Proband's response	Proband's response when she is not correct
128	191	forwards	pseudo	4	0	jets	stej	stej	
129	156	forwards	pseudo	4	0	geče	ečeg	ečeg	
130	77	forwards	low-freq	4	1	brka	akrb	akrb	
131	129	forwards	low-freq	4	1	keks	skek	skek	
132	173	forwards	low-freq	4	1	meče	ečem	ečem	
133	181	forwards	high-freq	4	157	kula	aluk	aluk	
134	171	forwards	high-freq	4	173	peti	itep	itep	
135	208	forwards	high-freq	4	180	sala	alas	alas	
136	38	forwards	pseudo	5	0	hapka	akpah	apkah	apkah
137	145	forwards	pseudo	5	0	nilet	telin	telin	
138	45	forwards	pseudo	5	0	gotap	patog	patog	
139	142	forwards	low-freq	5	1	čarka	akrač	akrač	
140	213	forwards	low-freq	5	1	cimet	temic	temic	
141	32	forwards	low-freq	5	1	dolap	palod	palod	
142	234	forwards	high-freq	5	170	roman	namor	namor	
143	178	forwards	high-freq	5	180	bogat	tagob	tagob	
144	29	forwards	high-freq	5	181	odelo	oledo	oledo	
145	168	forwards	pseudo	6	0	kofuda	adufok	adufok	
146	183	forwards	pseudo	6	0	polona	anolop	anolop	
147	187	forwards	pseudo	6	0	tamiti	itimat	itimat	
148	49	forwards	low-freq	6	1	moruna	anurom	anurom	
149	221	forwards	low-freq	6	1	tragač	čagart	čagart	
150	22	forwards	low-freq	6	1	unučad	dačunu	dačunu	
151	131	forwards	high-freq	6	170	zenica	acinez	acinez	
152	91	forwards	high-freq	6	182	dodati	itadod	itadod	
153	44	forwards	high-freq	6	182	gasiti	itisag	itisag	

No of stimuli	Order in which stimuli were presented	Forwards or Backwards	Low/High-Frequency- or Pseudo-words	Word length	Lemma frequency	Stimulus	Correct response	Proband's response	Proband's response when she is not correct
154	58	forwards	pseudo	7	0	lobiret	teribol	teribol	
155	118	forwards	pseudo	7	0	gofanak	kanafog	kanafog	
156	233	forwards	pseudo	7	0	fronela	alenorf	alenorf	
157	2	forwards	low-freq	7	1	oligarh	hragilo	ragilho	ragilho
158	121	forwards	low-freq	7	1	potanko	oknatop	oknatop	
159	117	forwards	low-freq	7	1	prečica	acičerp	acičerp	
160	192	forwards	high-freq	7	174	odličan	načildo	načildo	
161	125	forwards	high-freq	7	180	apoteka	aketopa	aketopa	
162	18	forwards	high-freq	7	186	promena	anemorp	anemorp	
163	163	forwards	pseudo	8	0	ozletalo	olatelzo	olatelzo	
164	36	forwards	pseudo	8	0	ginovati	itavonig	itavogin	itavogin
165	227	forwards	pseudo	8	0	tazomati	itamozat	itamozat	
166	138	forwards	low-freq	8	1	klasično	ončisalk	ončisalk	
167	113	forwards	low-freq	8	1	mauzolej	jelozuam	leuzojam	leuzojam
168	66	forwards	low-freq	8	1	nabasati	itasaban	itasaban	
169	114	forwards	high-freq	8	170	ispuniti	itinupsi	itinupsi	
170	205	forwards	high-freq	8	178	radovati	itavodar	itavodar	
171	179	forwards	high-freq	8	186	milovati	itavolim	itavolim	
172	3	forwards	pseudo	9	0	inumacija	ajicamuni	ajicamuni	
173	202	forwards	pseudo	9	0	čikovetar	ratevokič	ratevokič	
174	130	forwards	pseudo	9	0	šanolakaj	jakalonaš	jakalonaš	
175	116	forwards	low-freq	9	1	brodarica	aciradorb	aciradorb	
176	67	forwards	low-freq	9	1	intuicija	ajiciutni	ajiciutni	
177	219	forwards	low-freq	9	1	natalitet	tetilatan	tetilana	tetilana
178	174	forwards	high-freq	9	152	devojčica	acičjoved	ajicčoved	ajicčoved
179	52	forwards	high-freq	9	186	saobraćaj	jaćarboas	jaćarboas	

No of stimuli	Order in which stimuli were presented	Forwards or Backwards	Low/High-Frequency- or Pseudo-words	Word length	Lemma frequency	Stimulus	Correct response	Proband's response	Proband's response when she is not correct
180	105	forwards	high-freq	9	192	kilometar	ratemolik	ratemolik	
181	60	forwards	pseudo	10	0	bogasibati	itabisagob	itabisogap	itabisogap
182	188	forwards	pseudo	10	0	hetmozopan	napozomteh	napozomteh	
183	140	forwards	pseudo	10	0	otkobalati	italabokto	itabolotkoo	itabolokto
184	81	forwards	low-freq	10	1	zgradurina	anirudargz	anirudargz	
185	7	forwards	low-freq	10	1	razasipati	itapisazar	itipasizar	itipasizar
186	153	forwards	low-freq	10	1	rafinirano	onarinifar	oranifiran	oranifiran
187	41	forwards	high-freq	10	140	zaustaviti	itivatsuaz	ivatsuaz	ivatsuaz
188	194	forwards	high-freq	10	196	prostorija	ajirotsorp	ajirotsorp	
189	50	forwards	high-freq	10	209	jednosoban	nabosondej	nabosondej	
190	195	forwards	pseudo	11	0	apalitanski	iksnatilapa	iksnatipilan	iksnatipilan
191	150	forwards	pseudo	11	0	zokimanizam	mazinamikoz	mazohizam	mazohizam
192	136	forwards	pseudo	11	0	lugomonilac	calinomogul	calonigomul	calonigomul
193	100	forwards	low-freq	11	1	antagonizam	mazinogatna	mazogitna	mazogitna
194	73	forwards	low-freq	11	1	ispreturati	itaruterpsi	itaruterpsi	
195	5	forwards	low-freq	11	1	liberalizam	mazilarebil	mazilarebil	
196	103	forwards	high-freq	11	100	temperatura	arutarepmet	arutapmerter	arutapmerter
197	97	forwards	high-freq	11	209	rukovodilac	calidovokur	cavolidukur	cavolidukur
198	33	forwards	high-freq	11	220	socijalizam	mazilajicos	maizalicos	maizalicos
199	4	forwards	pseudo	12	0	olkaminacija	ajicanimaklo	ajicanikumo	ajicanikumo
200	112	forwards	pseudo	12	0	nimidrapstvo	ovtspardimin	ostvariti	ostvariti
201	101	forwards	pseudo	12	0	zehledalimat	tamiladelhez	tamiledhez	tamiledhez
202	20	forwards	low-freq	12	1	pravougaonik	kinoaguovarp	kinavoougarp	kinavoougarp
203	115	forwards	low-freq	12	1	stimulativan	navitalumits	navitulimps	navitulimps
204	232	forwards	low-freq	12	1	fermentacija	ajicatnemref	ajicatenemref	ajicatenemref
205	157	forwards	high-freq	12	20	kanalizacija	ajicazilanak	ajicazilinak	ajicazilinak

No of stimuli	Order in which stimuli were presented	Forwards or Backwards	Low/High-Frequency- or Pseudo-words	Word length	Lemma frequency	Stimulus	Correct response	Proband's response	Proband's response when she is not correct
206	119	forwards	high-freq	12	23	laboratorija	ajirotarobal	ajitarobal	ajitarobal
207	120	forwards	high-freq	12	903	organizacija	ajicazinagro	ajicanizirgo	ajicanizirgo
208	167	forwards	pseudo	13	0	balranemdalan	naladmenarlab	naladmeralab	naladmeralab
209	90	forwards	pseudo	13	0	olkamizađioni	inoiđazimaklo	inoziađiolko	inoziađiolko
210	143	forwards	pseudo	13	0	namidezdacija	ajicadzediman	ajitsadzineman	ajitsadzineman
211	144	forwards	low-freq	13	1	eksplikativan	navitakilpske	natsitalipkske	natsitalipkske
212	8	forwards	low-freq	13	1	meteorologija	ajigoloroetem	ajigoloroetem	
213	200	forwards	low-freq	13	1	orijentaciono	onoicatnejiro	oniacorinjetso	oniacorinjetsko
214	14	forwards	high-freq	13	36	kvalifikacija	ajicakifilavk	ajicakilifavk	ajicakilifalfk
215	71	forwards	high-freq	13	52	manifestacija	ajicatsefinam	ajicefacinam	ajicefacinam
216	26	forwards	high-freq	13	52	organizacioni	inoicazinagro	inocaizorgo	inocaizorgo
217	80	forwards	pseudo	14	0	lebrezembakija	ajikabmezerbel	NEXT	NEXT
218	110	forwards	pseudo	14	0	atnimiklamcija	ajicmalkiminta	ajicakilipmapab	ajicakilipmapab
219	88	forwards	pseudo	14	0	teolkamitacija	ajicatimakloet	ajicakilteopok	ajicakilteopok
220	24	forwards	low-freq	14	1	ekspresionista	atsinoiserpske	atsoinerkspe	atsoinerkspe
221	28	forwards	low-freq	14	1	velikoposednik	kindesopokilev	kindesopokilev	
222	162	forwards	low-freq	14	1	naturalizacija	ajicazilarutan	ajicazurilitan	ajicazurilitan
223	86	forwards	high-freq	14	19	interpretacija	ajicaterpretni	ajicaterpretni	
224	94	forwards	high-freq	14	27	zainteresovati	itavoseretniaz	itasovoeretsni	itasovoeretsni
225	141	forwards	high-freq	14	57	reorganizacija	ajicazinagroer	ajicanozorgioer	ajicanozorgioer
226	198	forwards	pseudo	15	0	arminizdrapilan	nalipardzinimra	NEXT	NEXT
227	111	forwards	pseudo	15	0	lebrezendaminac	canimadnezerbel	canizarebmilda	canizarebmilda
228	124	forwards	pseudo	15	0	bastukobromstvo	ovtsmorbokutsab	ovtsnogultmab	ovtsnogultmab
229	164	forwards	low-freq	15	1	profesionalizam	mazilanoiseforp	mazilanoifesorp	mazilanoifesorp
230	108	forwards	low-freq	15	1	diskvalifikovan	navokifilavksid	navokilafhoksfid	navokilafhoksfid
231	214	forwards	low-freq	15	1	idolopoklonstvo	ovtsnolkopolodi	ovtsnodolodi	ovtsnodolodi

No of stimuli	Order in which stimuli were presented	Forwards or Backwards	Low/High-Frequency- or Pseudo-words	Word length	Lemma frequency	Stimulus	Correct response	Proband's response	Proband's response when she is not correct
232	215	forwards	high-freq	15	12	poslastičarnica	acinračitsalsop	acinračitsolap	acinračitsolap
233	199	forwards	high-freq	15	17	elektrotehnički	ikčinhetortkele	ikčinhetortkele	
234	17	forwards	high-freq	15	25	internacionalan	nalanoicanretni	nailaconretni	nailaconretni

Table S6. Lexical decision task reaction time of the proband and father.

	Proband		
	Forward-words	Backward-words	Pseudo-words
	RT (SD)	RT (SD)	RT (SD)
**Visual lexical decision task**	662 (170)	979 (164)*	982 (185)*
**Auditory lexical decision task**	874 (101)	1996 (577)#	1896 (541)#
	Father		
**Auditory lexical decision task**	523 (274)	1617 (540)#	1736 (412)#

Mean values of reaction time (RT) are given in milliseconds (ms) and standard deviations can be found in the brackets.

* significantly different from visual lexical decision task, forward condition

# significantly different from auditory lexical decision task, forward condition

Table S7. Error rates and processing latencies of the proband as observed in visual lexical decision task and auditory lexical decision task.

Visual lexical decision task - Processing Latency				
	Forward-words		Reverse-words	
	Abstract	Concrete	Abstract	Concrete
High frequency	609 (93)	596 (77)	1051 (177)	939 (143)
Low frequency	776 (277)	675 (114)	958 (125)	975 (196)

Visual lexical decision task - Error %				
	Forward-words		Reverse-words	
	Abstract	Concrete	Abstract	Concrete
High frequency	0	0	33.333	20
Low frequency	6.667	0	40	20

Auditory lexical decision task- Processing Latency				
	Forward-words		Reverse-words	
	Abstract	Concrete	Abstract	Concrete
High frequency	878 (83)	849 (111)	2001 (557)	1856 (503)
Low frequency	911 (133)	857 (71)	2279 (620)	1943 (625)

Auditory lexical decision task- Error %				
	Forward-words		Reverse-words	
	Abstract	Concrete	Abstract	Concrete
High frequency	0	13.333	13.333	0
Low frequency	13.333	6.667	33.333	0

Processing latencies are given in milliseconds (ms) and standard deviations can be found in the brackets. Error is given as % responses incorrect.

Table S8. Results of the analyses of errors and response latencies of the proband as observed in visual lexical decision task and auditory lexical decision task.

Visual lexical decision task – Response latencies				
	Coefficient	Std Error	t	P (>|t|)
Intercept	6.8845	0.0394	174.92	<0.0001
Grapheme/phoneme sequence (Forward)	-0.4928	0.0518	-9.51	<0.0001
Frequency (Low)	-0.023	0.0563	-0.41	0.6844
Grapheme/phoneme sequence (Forward) x Frequency (Low)	0.1834	0.074	2.48	0.015
Visual lexical decision task – Errors (%)				
	Coefficient	Std Error	Wald Z	P(>|Z|)
Intercept	0.928	0.2865	3.24	0.0012
Grapheme/phoneme sequence (Forward)	3.1495	1.0483	3	0.0027
Auditory lexical decision task – Response Latencies				
	Coefficient	Std Error	t	P(>|t|)
Intercept	7.5591	0.0295	256.66	<0.0001
Grapheme/phoneme sequence (Forward)	-0.7929	0.0413	-19.21	<0.0001
Auditory lexical decision task – Errors (%)				
	Coefficient	Std Error	Wald Z	P(>|Z|)
Intercept	1.7346	0.3616	4.8	<0.0001
Concreteness (Concrete)	1.6327	0.805	2.03	0.0425

For each analysis the logistic regression coefficient, standard error value (Std Error), t-value (t) or Wald Z score (Wald Z), and P value are given.

Table S9. Coefficients from logistic regression model of control group's (n=6) accuracy in dual task.

	Coefficient	Std error	Wald Z	Pr(>|Z|)
Intercept (Test condition: Baseline, Word direction: Backward)	-0.0533	0.1634	-0.33	0.744
Test condition (Visual matrices)	0.0800	0.231	0.35	0.729
Test condition (Random number generation)	-1.2918	0.2595	-4.98	<0.0001
Test condition (Number series)	-0.7004	0.2394	-2.93	0.0034
Test condition (Num-pad sequence)	-1.6553	0.2794	-5.93	<0.0001
Test condition (Corsi block tapping task)	-0.0534	0.2311	-0.23	0.8172
Test condition (de-da)	-1.3329	0.2614	-5.1	<0.0001
Word direction (Forward)	3.6507	0.5325	6.86	<0.0001
Test condition (Visual matrices) x Word direction (Forward)	-0.4993	0.6956	-0.72	0.4729
Test condition (Random number generation) x Word direction (Forward)	-2.0644	0.5927	-3.48	0.0005
Test condition (Number series) x Word direction (Forward)	-0.8398	0.6169	-1.36	0.1734
Test condition (Num-pad sequence) x Word direction (Forward)	-0.896	0.6079	-1.47	0.1405
Test condition (Corsi block tapping task) x Word direction (Forward)	-1.1015	0.6331	-1.74	0.0819
Test condition (de-da) x Word direction (Forward)	-0.6549	0.6109	-1.07	0.2837

For each analysis the logistic regression coefficient, standard error value (Std Error), Wald Z score (Wald Z), and P value (Pr(>|Z|) are given.

Table S10. Coefficients from logistic regression model for the accuracy of the proband in dual task.

	Coef.	Std error	Wald Z	Pr(>|Z|)
Intercept (Test condition: Baseline, Word direction: Backward)	1.9924	0.6155	3.24	0.0012
Test condition (Visual matrices)	-1.7513	0.7356	-2.38	0.0173
Test condition (Random number generation)	-3.9849	0.8704	-4.58	<0.0001
Test condition (Number series)	-1.5870	0.7385	-2.15	0.0317
Test condition (Num-pad sequence)	-4.4348	0.9603	-4.62	<0.0001
Test condition (Corsi block tapping task)	-1.7513	0.7356	-2.38	0.0173
Test condition (de-da)	-1.9124	0.7342	-2.6	0.0092
Word direction (Forward)	1.1856	1.1918	0.99	0.3198
Test condition (Visual matrices) x Word direction (Forward)	1.0156	1.4582	0.7	0.4861
Test condition (Random number generation) x Word direction (Forward)	0.7268	1.3998	0.52	0.6036
Test condition (Number series) x Word direction (Forward)	8.7585	35.3748	0.25	0.8045
Test condition (Num-pad sequence) x Word direction (Forward)	1.4979	1.4582	1.03	0.3043
Test condition (Corsi block tapping task) x Word direction (Forward)	8.9228	35.3748	0.25	0.8009
Test condition (de-da) x Word direction (Forward)	9.0839	35.3747	0.26	0.7973

For each analysis the logistic regression coefficient, standard error value (Std Error), Wald Z score (Wald Z), and P value (Pr(>|Z|) are given.

Table S11. Proband: fMRI coordinates for areas active in forward- or backward-condition.

	Forward				Backward			
		MNI				MNI		
Area	z-score	*x*	*y*	*z*	z-score	*x*	*y*	*z*
Supplementary motor area	5.32	-4	6	54	7.49	-4	6	54
Primary motor (left)	4.04	-52	4	16	7.29	-52	-6	28
Primary motor (right)	NA	/	/	/	4.41	58	-4	30
Parietal (left)	5.50	-28	-58	38	9.19	-22	-72	54
Occipital	4.74	4	-88	26	5.99	-24	-82	22
Inferior frontal gyrus (left)	4.38	-58	6	12	6.32	-52	4	16
Prefrontal area	3.68	-38	30	34	4.72	-32	34	36
Prefrontal gyrus	NA	/	/	/	4.86	-44	30	4
Superior temporal gyrus (left)	6.01	-50	-44	22	8.08	-50	-42	22
Medial temporal posterior (left)	4.45	-54	-58	6	5.92	-52	-58	2
Fusiform gyrus (left)	NA	/	/	/	7.20	-42	-72	-14
Cerebellum (right)	3.19	34	-74	-26	5.94	36	-70	-30
Cerebellum (left)	2.91	-46	-64	-24	2.80	-40	-48	-34

*NA – not available

Table S12. Proband: fMRI coordinates for areas highly active only in forward- or backward-condition.

	Forward-"speech" – Backward-"speech"			
			**MNI**	
**Area**	**z-score**	**X**	**Y**	**Z**
Parietal cortex (left)	4.58	-50	-64	40
Parietal cortex (right)	3.04	54	-54	46
Frontal cortex (medial)	3.07	-2	54	-4
Anterior cingulate cortex	3.83	-2	40	14
Posterior cingulate cortex	3.88	-10	-46	34
	Backward-"speech" – Forward-"speech"			
			**MNI**	
**Area**	**z-score**	**X**	**Y**	**Z**
Parietal cortex (left)	4.62	-36	-44	52
Prefrontal cortex (left)	3.96	-44	30	6
Primary motor area (left)	4.60	-46	-12	32
Inferior frontal gyrus (left)	4.62	-58	-4	18
Supramarginal gyrus (left)	3.84	-60	-34	32
Superior temporal gyrus (left)	3.82	-58	-40	26
Fusiform gyrus (left)	4.65	-46	-72	-14
Primary motor area (right)	3.96	52	-4	28
Inferior frontal gyrus (right)	4.74	60	0	16

The areas significantly more active in the forward and backward-"speech" conditions are shown in the upper and bottom part of the table, respectively. The corresponding Z-scores and MNI coordinates are shown next to the area name.

Table S13. Exome sequencing metrics

Lane Metrics

Lane	Length	Tiles	Clusters	% clusters that passed filters	Yield (Mb)	Yield (Mb Q20)	% Mapped	% Coverage (genome)	% Primer	% Broken	% Variants	Hets
1	100	96	2487665	93.1	21839.52	21175.27	92.2	12.5	0	4.9	0.39 ± 0.00	0.0027

Lane	Mean coverage	% high coverage	% dups	% pair dups	% GC	% GC_{mapped}	% exonic	% exon coverage	%low $Quality_{end}$	avgQuality
1	8.59	5.65	27.66	15.62	46.3 ± 9.7	46.4 ± 10.1	31.9	98.7	0	35.9

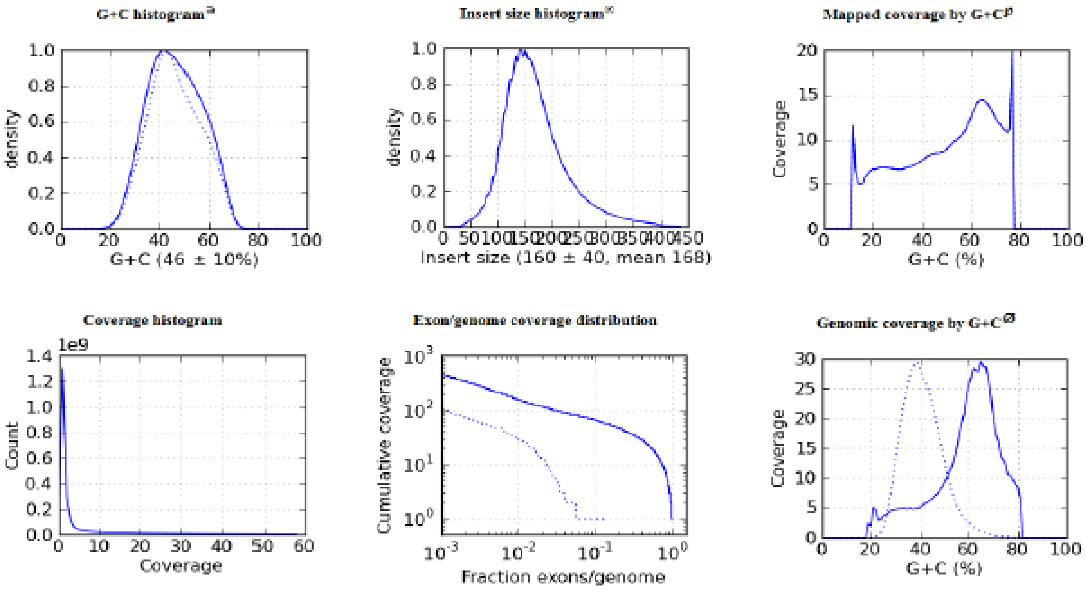

Sample Metrics

Sample name	Mean cvg	Median cvg	% > 10X	% > 20X	Exonic mean coverage	Exonic median coverage	Exonic % > 10X	Exonic % > 20X
Father	4.24	1	1.6	0.7	13.88	11	56.3	24.2
Daughter 1	8.45	1	2.9	2.1	38.74	35	87.6	75
Daughter 2	6.37	1	2.4	1.4	22.76	20	80.1	51.7
Cousin 1	9.82	1	3.1	2.3	47.74	44	89.8	80.6
Cousin 2	3.55	1	1.4	0.5	11.81	9	46.6	17.9
Mother	6.86	1	2.8	2	34.89	31	88.2	71.6
Average	6.55	1.00	2.37	1.50	28.30	25.00	74.77	53.50

Table S14. Predicted effects of shared variants of putative significance that validated.

Variant (chr:posn) (hg19)	Gene	Ref	Variant	exon: cDNAchange: AAChange	Poly Phen2 HDIV	Poly Phen2 HVAR	LRT	MutationTaster	Mutation Assessor	PhyloP	Phast Cons	SIFT
g.11:8161603	*RIC3*	C	T	exon2: c.G262A: p.G88R	**0.999**	**0.974**	0.003	**0.514**	**1.995**	**2.625**	**597**	**0.03**
g.6:3105973	*RIPK1*	C	G	exon8: c.C1264G: p.Q422E	0.082	0.036	0.119	.	1.78	**2.641**	**476**	0.18
g.15:28491062	*HERC2*	T	A	exon23: c.A3542T: p.D1181V	**0.999**	**0.996**	**0.000**	**0.999**	1.61	**2.274**	**559**	0.38
g.11:10874596	*ZBED5*	G	A	exon3: c.C1897T: p.R633C	**1.000**	**0.967**	.	.	1.895	**1.168**	**731**	**0.01**

Predicted effects that are significant are shown in bold

Table S15. UbPred Predicitions

Gene	Isoform	Residue	Wild-type sequence		Variant Sequence		
			**Score**	**Ubiquitinated?**	**Score**	**Ubiquitinated?**	**Δ score**
ZBED5	NP_067034	24	0.55	No	0.55	No	0
ZBED5	NP_067034	41	0.64	Low confidence	0.64	Low confidence	0
ZBED5	NP_067034	46	0.58	No	0.59	No	0.01
ZBED5	NP_067034	64	0.94	High confidence	0.92	High confidence	0.02
ZBED5	NP_067034	73	0.74	Medium confidence	0.73	Medium confidence	0.01
ZBED5	NP_067034	81	0.91	High confidence	0.91	High confidence	0
ZBED5	NP_067034	82	0.86	High confidence	0.86	High confidence	0
ZBED5	NP_067034	91	0.31	No	0.32	No	0.01
ZBED5	NP_067034	98	0.16	No	0.18	No	0.02
ZBED5	NP_067034	103	0.08	No	0.1	No	0.02
ZBED5	NP_067034	104	0.1	No	0.1	No	0
ZBED5	NP_067034	106	0.15	No	0.14	No	0.01
ZBED5	NP_067034	109	0.39	No	0.39	No	0
ZBED5	NP_067034	136	0.38	No	0.39	No	0.01
ZBED5	NP_067034	137	0.34	No	0.34	No	0
ZBED5	NP_067034	148	0.14	No	0.14	No	0
ZBED5	NP_067034	156	0.21	No	0.23	No	0.02
ZBED5	NP_067034	161	0.45	No	0.45	No	0
ZBED5	NP_067034	163	0.79	Medium confidence	0.77	Medium confidence	0.02
ZBED5	**NP_067034**	**169**	**0.82**	**Medium confidence**	**0.85**	**High confidence**	**0.03**
ZBED5	NP_067034	179	0.89	High confidence	0.89	High confidence	0
ZBED5	**NP_067034**	**184**	**0.88**	**High confidence**	**0.81**	**Medium confidence**	**0.07**
ZBED5	NP_067034	219	0.42	No	0.37	No	0.05
ZBED5	NP_067034	223	0.44	No	0.43	No	0.01
ZBED5	NP_067034	236	0.54	No	0.54	No	0
ZBED5	NP_067034	237	0.45	No	0.44	No	0.01
ZBED5	NP_067034	253	0.53	No	0.55	No	0.02
ZBED5	NP_067034	268	0.41	No	0.42	No	0.01
ZBED5	NP_067034	299	0.55	No	0.54	No	0.01
ZBED5	NP_067034	330	0.5	No	0.51	No	0.01
ZBED5	NP_067034	337	0.48	No	0.41	No	0.07
ZBED5	NP_067034	352	0.48	No	0.52	No	0.04
ZBED5	NP_067034	361	0.42	No	0.41	No	0.01
ZBED5	NP_067034	382	0.3	No	0.31	No	0.01
ZBED5	NP_067034	389	0.37	No	0.39	No	0.02
ZBED5	NP_067034	403	0.16	No	0.11	No	0.05
ZBED5	NP_067034	413	0.43	No	0.41	No	0.02
ZBED5	NP_067034	439	0.21	No	0.22	No	0.01
ZBED5	NP_067034	483	0.41	No	0.43	No	0.02
ZBED5	NP_067034	494	0.64	Low confidence	0.64	Low confidence	0
ZBED5	NP_067034	504	0.42	No	0.44	No	0.02
ZBED5	NP_067034	511	0.36	No	0.4	No	0.04
ZBED5	NP_067034	543	0.57	No	0.59	No	0.02
ZBED5	NP_067034	562	0.45	No	0.46	No	0.01
ZBED5	NP_067034	583	0.61	No	0.55	No	0.06
ZBED5	**NP_067034**	**605**	**0.69**	**Medium confidence**	**0.68**	**Low confidence**	**0.01**
ZBED5	NP_067034	655	0.35	No	0.38	No	0.03
ZBED5	NP_067034	657	0.33	No	0.34	No	0.01
ZBED5	NP_067034	660	0.12	No	0.13	No	0.01
ZBED5	NP_067034	680	0.11	No	0.11	No	0
ZBED5	NP_067034	685	0.07	No	0.07	No	0
ZBED5	NP_067034	686	0.07	No	0.07	No	0
ZBED5	NP_067034	689	0.13	No	0.12	No	0.01

		Wild-type sequence			Variant Sequence		
**Gene**	**Isoform**	**Residue**	**Score**	**Ubiquitinated?**	**Score**	**Ubiquitinated?**	**Δ score**
RIPK1	NP_003795	11	0.58	No	0.58	No	0
RIPK1	NP_003795	13	0.52	No	0.59	No	0.07
RIPK1	NP_003795	30	0.46	No	0.48	No	0.02
RIPK1	NP_003795	45	0.37	No	0.38	No	0.01
RIPK1	NP_003795	49	0.33	No	0.41	No	0.08
RIPK1	NP_003795	65	0.49	No	0.46	No	0.03
RIPK1	NP_003795	77	0.28	No	0.29	No	0.01
RIPK1	NP_003795	87	0.38	No	0.4	No	0.02
RIPK1	NP_003795	97	0.44	No	0.44	No	0
RIPK1	NP_003795	105	0.44	No	0.4	No	0.04
RIPK1	NP_003795	115	0.25	No	0.27	No	0.02
RIPK1	NP_003795	132	0.31	No	0.31	No	0
RIPK1	NP_003795	137	0.36	No	0.37	No	0.01
RIPK1	NP_003795	140	0.42	No	0.44	No	0.02
RIPK1	NP_003795	153	0.32	No	0.31	No	0.01
RIPK1	NP_003795	163	0.34	No	0.3	No	0.04
RIPK1	NP_003795	167	0.61	No	0.54	No	0.07
RIPK1	**NP_003795**	**184**	**0.66**	**Low confidence**	**0.69**	**Medium confidence**	**0.03**
RIPK1	**NP_003795**	**185**	**0.68**	**Low confidence**	**0.7**	**Medium confidence**	**0.02**
RIPK1	**NP_003795**	**204**	**0.69**	**Medium confidence**	**0.63**	**Low confidence**	**0.06**
RIPK1	NP_003795	208	0.5	No	0.5	No	0
RIPK1	NP_003795	225	0.33	No	0.32	No	0.01
RIPK1	NP_003795	242	0.28	No	0.25	No	0.03
RIPK1	NP_003795	265	0.4	No	0.39	No	0.01
RIPK1	NP_003795	284	0.57	No	0.5	No	0.07
RIPK1	**NP_003795**	**302**	**0.7**	**Medium confidence**	**0.66**	**Low confidence**	**0.04**
RIPK1	NP_003795	305	0.64	Low confidence	0.63	Low confidence	0.01
RIPK1	NP_003795	306	0.67	Low confidence	0.68	Low confidence	0.01
RIPK1	NP_003795	316	0.81	Medium confidence	0.8	Medium confidence	0.01
RIPK1	NP_003795	377	0.94	High confidence	0.86	High confidence	0.08
RIPK1	NP_003795	396	0.27	No	0.22	No	0.05
RIPK1	NP_003795	435	0.84	High confidence	0.88	High confidence	0.04
RIPK1	NP_003795	530	0.53	No	0.57	No	0.04
RIPK1	NP_003795	565	0.72	Medium confidence	0.78	Medium confidence	0.06
RIPK1	**NP_003795**	**571**	**0.71**	**Medium confidence**	**0.66**	**Low confidence**	**0.05**
RIPK1	**NP_003795**	**585**	**0.62**	**Low confidence**	**0.6**	**No**	**0.02**
RIPK1	NP_003795	596	0.17	No	0.13	No	0.04
RIPK1	NP_003795	599	0.15	No	0.14	No	0.01
RIPK1	NP_003795	604	0.33	No	0.3	No	0.03
RIPK1	NP_003795	625	0.59	No	0.55	No	0.04
RIPK1	NP_003795	627	0.52	No	0.46	No	0.06
RIPK1	NP_003795	634	0.19	No	0.16	No	0.03
RIPK1	NP_003795	642	0.35	No	0.35	No	0
RIPK1	NP_003795	648	0.34	No	0.32	No	0.02
RIC3	NP_078833	25	0.41	No	0.41	No	0
RIC3	NP_078833	32	0.62	Low confidence	0.62	Low confidence	0
RIC3	NP_078833	43	0.87	High confidence	0.87	High confidence	0
RIC3	**NP_078833**	**78**	**0.7**	**Medium confidence**	**0.41**	**No**	**0.29**
RIC3	NP_078833	80	0.68	Low confidence	0.66	Low confidence	0.02
RIC3	NP_078833	117	0.31	No	0.3	No	0.01
RIC3	NP_078833	120	0.3	No	0.28	No	0.02
RIC3	NP_078833	122	0.46	No	0.46	No	0
RIC3	NP_078833	129	0.77	Medium confidence	0.77	Medium confidence	0
RIC3	NP_078833	141	0.7	Medium confidence	0.7	Medium confidence	0
RIC3	**NP_078833**	**153**	**0.69**	**Medium confidence**	**0.68**	**Low confidence**	**0.01**

		Wild-type sequence			Variant Sequence		
**Gene**	**Isoform**	**Residue**	**Score**	**Ubiquitinated?**	**Score**	**Ubiquitinated?**	**Δ score**
RIC3	NP_078833	155	0.67	Low confidence	0.67	Low confidence	0
RIC3	NP_078833	163	0.66	Low confidence	0.64	Low confidence	0.02
RIC3	NP_078833	184	0.56	No	0.56	No	0
RIC3	NP_078833	198	0.34	No	0.34	No	0
RIC3	NP_078833	201	0.46	No	0.46	No	0
RIC3	NP_078833	210	0.7	Medium confidence	0.7	Medium confidence	0
RIC3	NP_078833	239	0.56	No	0.56	No	0
RIC3	NP_078833	253	0.77	Medium confidence	0.77	Medium confidence	0
RIC3	NP_078833	298	0.72	Medium confidence	0.72	Medium confidence	0
RIC3	NP_078833	334	0.82	Medium confidence	0.82	Medium confidence	0
RIC3	NP_078833	342	0.76	Medium confidence	0.76	Medium confidence	0
RIC3	NP_078833	352	0.75	Medium confidence	0.75	Medium confidence	0
RIC3	NP_078833	361	0.38	No	0.38	No	0
RIC3	NP_001193600	25	0.41	No	0.41	No	0
RIC3	**NP_001193600**	**32**	**0.62**	**Low confidence**	**0.61**	**No**	**0.01**
RIC3	NP_001193600	43	0.89	High confidence	0.88	High confidence	0.01
RIC3	**NP_001193600**	**78**	**0.70**	**Medium confidence**	**0.43**	**No**	**0.27**
RIC3	NP_001193600	80	0.68	Low confidence	0.65	Low confidence	0.03
RIC3	NP_001193600	117	0.31	No	0.31	No	0
RIC3	NP_001193600	120	0.29	No	0.27	No	0.02
RIC3	NP_001193600	122	0.45	No	0.47	No	0.02
RIC3	NP_001193600	129	0.77	Medium confidence	0.77	Medium confidence	0
RIC3	NP_001193600	141	0.70	Medium confidence	0.69	Medium confidence	0.01
RIC3	NP_001193600	153	0.73	Medium confidence	0.73	Medium confidence	0
RIC3	NP_001193600	155	0.66	Low confidence	0.67	Low confidence	0.01
RIC3	NP_001193600	163	0.78	Medium confidence	0.78	Medium confidence	0
RIC3	NP_001193600	185	0.56	No	0.57	No	0.01
RIC3	NP_001193600	199	0.34	No	0.34	No	0
RIC3	NP_001193600	202	0.51	No	0.51	No	0
RIC3	NP_001193600	211	0.76	Medium confidence	0.76	Medium confidence	0
RIC3	NP_001193600	240	0.55	No	0.55	No	0
RIC3	NP_001193600	254	0.77	Medium confidence	0.77	Medium confidence	0
RIC3	NP_001193600	299	0.72	Medium confidence	0.71	Medium confidence	0.01
RIC3	NP_001193600	335	0.82	Medium confidence	0.82	Medium confidence	0
RIC3	NP_001193600	343	0.76	Medium confidence	0.76	Medium confidence	0
RIC3	NP_001193600	353	0.75	Medium confidence	0.75	Medium confidence	0
RIC3	NP_001193600	362	0.38	No	0.38	No	0
RIC3	NP_001193601	25	0.43	No	0.43	No	0
RIC3	NP_001193601	32	0.65	Low confidence	0.65	Low confidence	0
RIC3	NP_001193601	43	0.87	High confidence	0.87	High confidence	0
RIC3	**NP_001193601**	**78**	**0.65**	**Low confidence**	**0.39**	**No**	**0.26**
RIC3	**NP_001193601**	**80**	**0.63**	**Low confidence**	**0.59**	**No**	**0.04**
RIC3	NP_001193601	117	0.26	No	0.26	No	0
RIC3	NP_001193601	120	0.28	No	0.27	No	0.01
RIC3	NP_001193601	122	0.44	No	0.45	No	0.01
RIC3	NP_001193601	129	0.71	Medium confidence	0.71	Medium confidence	0
RIC3	NP_001193601	141	0.71	Medium confidence	0.73	Medium confidence	0.02
RIC3	NP_001193601	159	0.59	No	0.59	No	0
RIC3	NP_001193601	173	0.88	High confidence	0.88	High confidence	0
RIC3	NP_001193601	218	0.70	Medium confidence	0.70	Medium confidence	0
RIC3	NP_001193601	254	0.82	Medium confidence	0.82	Medium confidence	0
RIC3	NP_001193601	262	0.76	Medium confidence	0.76	Medium confidence	0
RIC3	NP_001193601	272	0.74	Medium confidence	0.74	Medium confidence	0
RIC3	NP_001193601	281	0.37	No	0.37	No	0

For each isoform of the gene, ubiquitination signal predictions were compared between the wild type sequence and the variant sequence observed in the backward-speech family. For each lysine residue, a ubiquitination score is given (where 1.0 is the highest likelihood of ubiquitination at this residue). The confidence of the residue being ubiquitinated is also given where lysines with a score ≥0.62 are likely to be ubiquitinated. Lysines with a score of between 0.62 and 0.69 are predicted to be ubiquitinated with a low level of confidence, lysines with a score of between 0.69 and 0.84 are predicted to be ubiquitinated with a medium level of confidence and lysines with a score of between 0.84 and 1.00 are predicted to be ubiquitinated with a high level of confidence. Sites at which the observed variants resulted in a prediction change are highlighted in bold. The majority of these changes occurred at lysine residues which were near the threshold of confidence intervals. The largest change was observed at residue 78 of RIC3, where the variant resulted in the abolishment of a predicted ubiquitination signal across all three isoforms (change in unbiquitination score (Δ score) ranged from 0,26 to 0.29.

Table S16. Ubiquitination burden analysis

exonic and splicing, UTR variants

Individual	No. Genic Variants	No. Genes*	No. Unique genes/individual	No. Variants in genes interacting with UBC* High confidence	No. Variants in genes interacting with UBC* Low confidence	No. unique genes interacting with UBC High confidence	No. unique genes interacting with UBC Low confidence
II.3 (father)	15602	15731	8306	5184	1498	2732	874
III.3 (proband)	27522	27741	11147	8828	2593	3569	1151
III.4 (sister)	25144	25355	10760	8242	2374	3462	1102
III.1 (cousin 1)	28820	29106	11427	9358	2766	3637	1203
III.2 (cousin 2)	10735	10827	6461	3603	1145	2118	715
II.4 (mother)	25214	25418	10815	8189	2396	3471	1114
**Total**	**133037**	**134178**	**58916**	**43404**	**12772**	**18989**	**6159**
			**TTEST (p)**	**0.89**	**0.87**	**0.97**	**0.91**

exonic and splicing variants

Individual	No. Genic Variants	No. Genes*	No. Unique genes/individual	No. Variants in genes interacting with UBC* High confidence	No. Variants in genes interacting with UBC* Low confidence	No. unique genes interacting with UBC High confidence	No. unique genes interacting with UBC Low confidence
II.3 (father)	10593	10654	6237	3370	939	1985	602
III.3 (proband)	18215	18306	8490	5587	1580	2625	822
III.4 (sister)	16732	16818	8181	5243	1451	2539	771
III.1 (cousin 1)	18645	18759	8664	5790	1607	2642	850
III.2 (cousin 2)	7302	7348	4736	2377	727	1503	481
II.4 (mother)	16756	16840	8216	5220	1467	2571	795
**Total**	**88243**	**88725**	**44524**	**27587**	**7771**	**13865**	**4321**
			**TTEST (p)**	**0.91**	**0.90**	**0.98**	**0.94**

exonic or exonic/splicing nonsynonymous variants							
				No. Variants in genes interacting with UBC*		No. unique genes interacting with UBC	
Individual	No. Genic Variants	No. Genes*	No. Unique genes/individual	High confidence	Low confidence	High confidence	Low confidence
II.3 (father)	4156	4189	3078	1074	359	804	274
III.3 (proband)	7881	7919	4875	2027	683	1275	452
III.4 (sister)	7079	7113	4577	1869	600	1204	400
III.1 (cousin 1)	8081	8128	4962	2114	685	1310	448
III.2 (cousin 2)	2794	2813	2179	763	260	572	198
II.4 (mother)	7058	7095	4589	1862	604	1228	412
**Total**	**37049**	**37257**	**24260**	**9709**	**3191**	**6393**	**2184**
			**TTEST (p)**	**0.87**	**0.94**	**0.90**	**0.99**

exonic or exonic/splicing frameshift variants							
				No. Variants in genes interacting with UBC*		No. unique genes interacting with UBC	
Individual	No. Genic Variants	No. Genes*	No. Unique genes/individual	High confidence	Low confidence	High confidence	Low confidence
II.3 (father)	460	462	441	165	44	160	42
III.3 (proband)	142	146	136	32	11	28	11
III.4 (sister)	196	198	186	56	14	53	14
III.1 (cousin 1)	129	129	124	25	12	23	12
III.2 (cousin 2)	325	325	312	123	42	115	40
II.4 (mother)	191	191	181	57	19	53	19
**Total**	**1443**	**1451**	**1380**	**458**	**142**	**432**	**138**
			**TTEST (p)**	**0.71**	**0.79**	**0.71**	**0.80**

splicing or exonic/splicing variants							
				No. Variants in genes interacting with UBC*		No. unique genes interacting with UBC	
Individual	No. Genic Variants	No. Genes*	No. Unique genes/individual	High confidence	Low confidence	High confidence	Low confidence
II.3 (father)	71	71	69	22	7	22	7
III.3 (proband)	110	112	109	30	8	30	8
III.4 (sister)	99	100	96	25	8	25	8
III.1 (cousin 1)	112	113	111	38	8	38	8
III.2 (cousin 2)	55	55	55	18	7	18	7
II.4 (mother)	89	89	85	27	5	27	5
**Total**	**536**	**540**	**525**	**160**	**43**	**160**	**43**
			**TTEST (p)**	**0.87**	**0.60**	**0.87**	**0.60**

exonic stop-gain or stop-loss variants							
				No. Variants in genes interacting with UBC*		No. unique genes interacting with UBC	
Individual	No. Genic Variants	No. Genes*	No. Unique genes/individual	High confidence	Low confidence	High confidence	Low confidence
II.3 (father)	27	27	27	3	0	3	0
III.3 (proband)	52	52	52	7	0	7	0
III.4 (sister)	50	50	47	8	0	6	0
III.1 (cousin 1)	53	53	52	8	2	7	2
III.2 (cousin 2)	26	26	26	4	1	4	1
II.4 (mother)	44	44	44	8	0	8	0
**Total**	**252**	**252**	**248**	**38**	**3**	**35**	**3**
			**TTEST (p)**	**0.49**	**0.22**	**0.64**	**0.22**

exonic and splicing RARE variants							
				no. Variants in genes interacting with UBC*		no. unique genes interacting with UBC	
Individual	No. Genic Variants	No. Genes*	No. Unique genes/individual	High confidence	Low confidence	High confidence	Low confidence
II.3 (father)	137	140	135	37	17	37	17
III.3 (proband)	265	268	253	77	24	75	24
III.4 (sister)	225	226	221	66	25	66	25
III.1 (cousin 1)	255	257	249	95	28	92	28
III.2 (cousin 2)	67	68	67	27	7	27	7
II.4 (mother)	226	227	219	80	19	79	18
**Total**	**1175**	**1186**	**1144**	**382**	**120**	**376**	**119**
			**TTEST (p)**	**0.72**	**0.90**	**0.71**	**0.87**

The number of observed sequence variants in genes known to interact with UBC were compared between family members with the ability to backward-speak (II.3 (father) and II.3 (proband)) and those family members without the ability (III.4, III.1, III.2, II.4). No significant differences in variant burden were observed across groups.
